# Recent Advances in Electrochemical-Based Silicon Production Technologies with Reduced Carbon Emission

**DOI:** 10.34133/research.0142

**Published:** 2023-05-18

**Authors:** Feng Tian, Zhongya Pang, Shen Hu, Xueqiang Zhang, Fei Wang, Wei Nie, Xuewen Xia, Guangshi Li, Hsien-Yi Hsu, Qian Xu, Xingli Zou, Li Ji, Xionggang Lu

**Affiliations:** ^1^ State Key Laboratory of Advanced Special Steel & Shanghai Key Laboratory of Advanced Ferrometallurgy & School of Materials Science and Engineering, Shanghai University, 99^#^ Shangda Road, Shanghai 200444, China.; ^2^ Center for Hydrogen Metallurgy Technology, Shanghai University, Shanghai 200444, China.; ^3^ State Key Laboratory of ASIC and System, School of Microelectronics,Fudan University, 220^#^ Handan Road, Shanghai 200433, China.; ^4^ School of Energy and Environment, Department of Materials Science and Engineering, City University of Hong Kong, Kowloon Tong, Hong Kong, China.

## Abstract

Sustainable and low-carbon-emission silicon production is currently one of the main focuses for the metallurgical and materials science communities. Electrochemistry, considered a promising strategy, has been explored to produce silicon due to prominent advantages: (a) high electricity utilization efficiency; (b) low-cost silica as a raw material; and (c) tunable morphologies and structures, including films, nanowires, and nanotubes. This review begins with a summary of early research on the extraction of silicon by electrochemistry. Emphasis has been placed on the electro-deoxidation and dissolution–electrodeposition of silica in chloride molten salts since the 21st century, including the basic reaction mechanisms, the fabrication of photoactive Si films for solar cells, the design and production of nano-Si and various silicon components for energy conversion, as well as storage applications. Besides, the feasibility of silicon electrodeposition in room-temperature ionic liquids and its unique opportunities are evaluated. On this basis, the challenges and future research directions for silicon electrochemical production strategies are proposed and discussed, which are essential to achieve large-scale sustainable production of silicon by electrochemistry.

## Introduction

The growing demand for low-carbon technologies has sparked a important change in global energy consumption, prompting an imminent transition from fossil fuels to renewable and sustainable energy sources [1–5]. In this regard, silicon is one of the most important and sustainable chemical elements due to its abundance in the earth’s crust [6,7]. Silicon-based technologies are essential to harvesting and utilizing sustainable energy sources, such as wind, solar, tidal, and geothermal energy [8]. Various silicon-based energy technologies have been developed and used for efficient energy production and fast storage/release. For example, in the photovoltaic (PV) industry, 90% of the global solar cell modules are Si-based [9–11]. Additionally, silicon-based nanostructures are considered critical anodic materials for achieving high-energy, long-endurance Li-ion batteries and are also being established in currently niche markets, such as electric cars [12–15]. There is no doubt that silicon-based technologies have always been and will continue to be the frontier in future development.

Silicon commonly exists in oxide (silica/SiO_2_) or silicate minerals; thus, sophisticated reduction and purification steps are unavoidable [16]. In this regard, the carbothermal reduction of silica was a chemical innovation of the 19th century and remained the dominant process as the first step in the current silicon production industry. The purity of the silicon obtained by this process is generally between 98% and 99%, called metallurgical grade silicon (MG-Si), which is not pure enough for PV or other semiconductive applications. To prepare higher-purity solar-grade (SoG-Si, 99.9999%, 6 N) and electronic-grade silicon (EG-Si, ≥99.9999999%, 9 N), metallurgical silicon is transformed into silanes that can be easily distilled, separated, and purified. The silanes are chemically reduced and purified via Siemens, Bayer, or fluidized bed reactor processes to ultra-high-purity silicon [17–19]. Unfortunately, the carbothermic reduction and chemical refining processes are polluted heavily, and the total heating electricity consumption is enormous. Consequently, developing alternative, environmentally friendly, low-cost, and efficient technologies remains important for silicon extraction-related research areas. This awareness has led to an interest in molten salt silicon electrochemistry as a promising alternative to conventional processes. The silicon electrochemical extraction strategy has great potential to outperform conventional processes in terms of (a) the utilization of electrons as reducing agents rather than hazardous chemicals, (b) being straightforward as far as infrastructure is concerned, (c) the advantages of designing various silicon components, and (d) the potential to reduce the cost of producing SoG-Si [20–22].

**Fig. 1.**
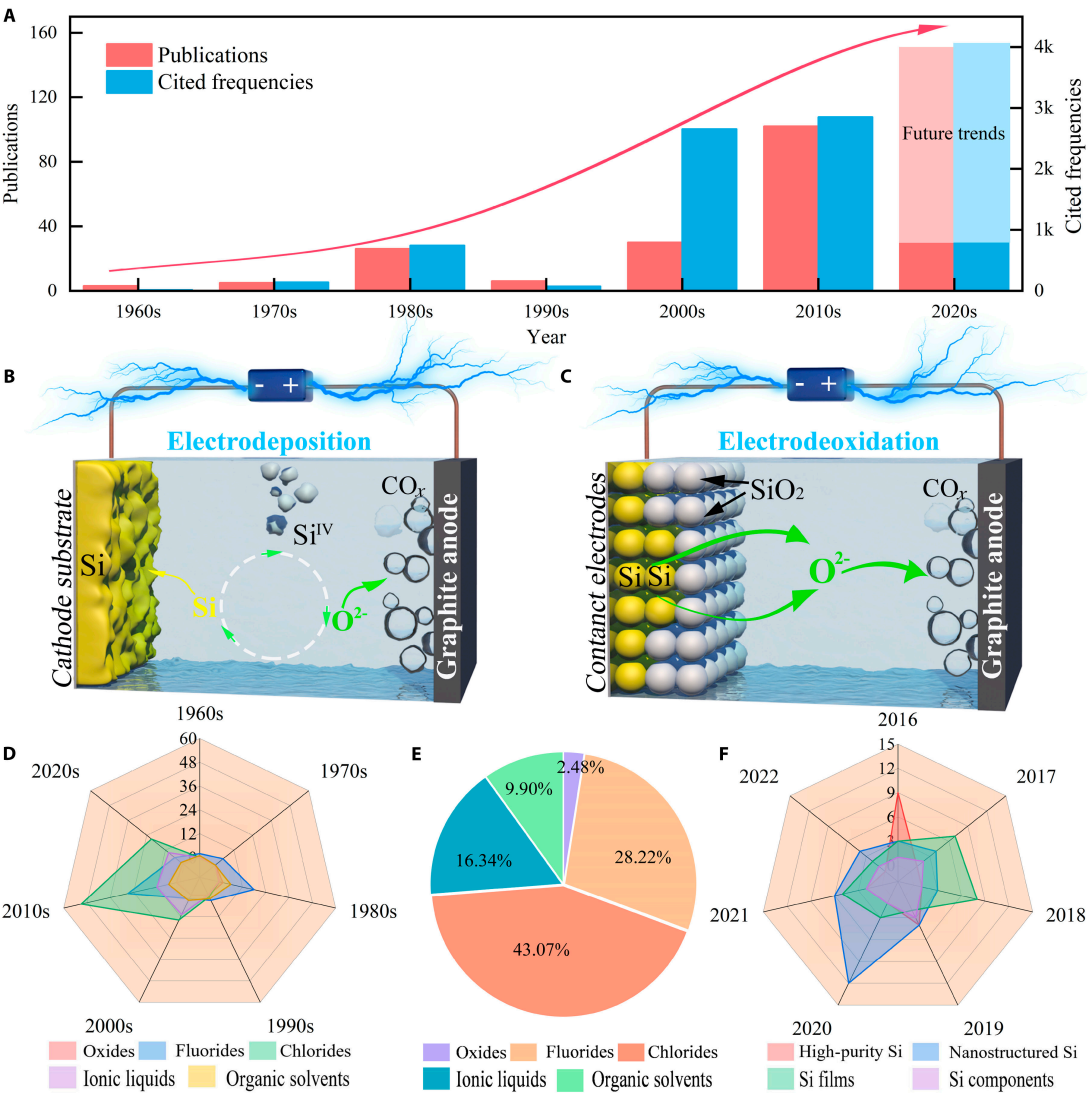
(A) The publication and citation numbers over the past 60 years with the search keywords of “Silicon & electrochemical reduction.” (These data originate from the Scopus database.) (B and C) Primary strategies for silicon production via electrochemistry: (B) electrodeposition and (C) electro-deoxidation. (D and E) Trends and comparison of scientific papers on electrochemical production of Si in different electrolytes. (F) Recent trends in the publication (number of papers) of various Si materials synthesized by electrochemistry.

Silicon electrochemistry has received tremendous attention in the last 2 decades because of its universal significance and current policy relevance in a landscape where countries around the world are advocating a low-carbon economy (Fig. 1A). Depending on the reaction mechanism, the electrochemical extraction procedure can be classified into electrodeposition and electro-deoxidation (Fig. 1B and C). Inspired by the Hall–Heroult process for aluminum electrowinning [23], early works focused on the electrolysis of sand in molten oxides and molten fluorides (Fig. 1D). This provided the opportunity to produce pure silicon at a cost comparable to that of aluminum. In 2003, a groundbreaking work demonstrated the direct reduction of solid silica by electrochemical “deoxidation” in molten chloride (CaCl_2_), launching a new era of silicon production by the electrolytic reduction of sand [24]. Accordingly, chloride salts (including CaCl_2_) are extensively investigated because of their eco-friendliness, water solubility, and low cost (Fig. 1E). Recently, silicon electrochemistry has shifted its focus toward the controlled fabrication of nanostructured silicon, silicon films, high-purity silicon, and Si components (Fig. 1F). These products offer a potentially cost-effective solution for industrial-scale energy applications because of the various advantages of the synthesis process: catalyst-free, template-free fabrication, and inexpensive feedstock. Additionally, ionic liquids offer new opportunities for the extraction of silicon by electrodeposition at low temperatures [25]. By now, several classic and outstanding reviews have been published to summarize significant advances in silicon electrolysis technology from various perspectives, including extraction of SoG-Si [26], fabrication of silicon-based anodes for energy storage [22,27], design of silicon surface structures [20,28], and development and recommendations for practical silicon production [21,29]. However, there is still a lack of a detailed review article with an extensive landscape, including the historical development, the current challenges, and the future pathways.

Here, we systematically review the historical progress and recent advances in the extraction of silicon by electrochemistry. The early silicon electro-reduction processes are briefly reviewed to capture the state of knowledge about half a century ago. The reaction system and mechanism, as well as the achievement of silicon extraction involved in these evolved silicon electrochemical strategies, are further discussed. Considering that CaCl_2_-based molten salt electrolysis offers a green and cost-effective way to synthesize new materials with various applications of silicon components, we emphatically summarize the electroreduction and electrodeposition of silicon materials in molten chlorides. Among them, the electroreduction strategy relying on solid–solid (SS) phase reaction and silicon electrodeposition process relying on solid–liquid–solid (SLS) reaction can be used to achieve electrochemical extraction and preparation of nanostructured silicon, high-purity silicon, and photo-responsive doped silicon. The electrochemical synthesis of silicides, silicon carbides (SiC), and silicon/carbon (Si/C) composites has been systematically reviewed. Furthermore, the silicon electrodeposition in room-temperature ionic liquids as a promising silicon extraction strategy has also been discussed and evaluated. More importantly, the current challenges and future directions of silicon electrochemistry are proposed and discussed in depth based on the existing process advances, with the expectation of contributing to the sustainable development of the silicon industry.

## Early Strategies and Development of Silicon Extraction by Electrochemistry

### Input from the electrolytic science and inspired by the Hall–Heroult process

**Fig. 2.**
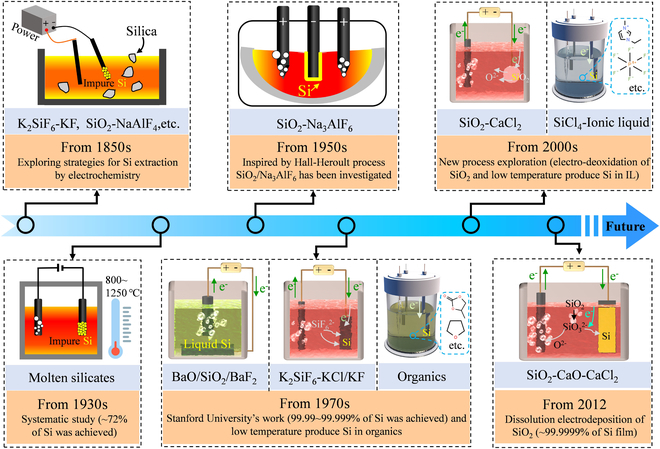
A timeline of the early development of silicon extraction by electrochemistry.

Electrolysis has been applied to obtain various metals since the invention of the first galvanic cells. Interestingly, the first extraction of silicon by electrochemical means dates back to the mid-19th century and has long been under investigation (Fig. 2), according to Elwell and colleagues [30,31]. The first recorded attempt was by Sainte-Claire Deville in 1854, who claimed that silicon was produced by electrolysis of impure NaAlCl_4_. The first silicon produced directly by electrolysis may be attributed to Ullik in 1865. In that experiment, silicon monomers were obtained from the electrolysis of K_2_SiF_6_ in molten KF. However, systematic research on the electrosynthesis of silicon was started in the 1930s. It is documented that researchers have carried out electrowinning of silicon in various molten silicates at 800 to 1,250 °C [31]. The maximum silicon content was ~72% due to the high applied potential (which also reduced alkali or alkaline earth metals).

Following these early explorations, in the early 1950s, there was a growing interest in exploring the direct electrolysis of sand oxide to extract high-purity silicon. The extraction of pure silicon products that can be applied owes much to the knowledge gained on the Hall–Heroult process for aluminum electrolysis developed in 1886, and many of the techniques and concepts established for it are still widely utilized to date. The primary feature of this process is the electrolysis of Al_2_O_3_ at a temperature above the melting point of aluminum. The used electrodes were made from glassy carbon, while cryolite was the solvent. The obtained aluminum was of high purity (99.999%). According to Faraday’s law (Eq. 1), the energy consumption required for 1 kg of aluminum produced can be calculated, where *F* is Faraday’s constant, *V* is the cell voltage, *z* is the valence of the metal, *A* is the atomic weight, and *η* is the cell efficiency. Substituting *z* = 3 and *A* = 27, we can obtain Eq. 2, and a typical cell voltage is about 4 V and *η* = 90%, so the power required to produce 1 kg of Al by electrolysis of Al_2_O_3_ is about 47 MJ kg^−1^ (or expressed as 13.06 kWh kg^−1^). This value is considered to be widely accepted. Correspondingly, in the case of silicon electrolysis, *z* = 4 and *A* = 28 in Eq. 1, so Eq. 3 is obtained, and assuming that the values of *V* and *η* for silicon electrolysis are approximately the same as for aluminum electrolysis, 28.5% more electrical energy is required compared to the aluminum electrolysis process, which is 16.7 kWh kg^−1^.

$$W=\frac{\mathrm{FVz}}{A\eta }$$ (1)

$$W=\frac{10.73V}{\eta }\mathrm{MJ}\;{\mathrm{kg}}^{–1}$$ (2)

$$W=\frac{13.79V}{\eta }\mathrm{MJ}\;{\mathrm{kg}}^{–1}$$ (3)

SiO_2_ used in silicon electrolysis is cheaper and more accessible than the starting material for aluminum electrolysis. This cost reduction via the raw material equals the pretreatment consumption of raw materials for aluminum electrolysis, resulting in a slight difference in cost between silicon electrolysis and aluminum electrolysis. Thus, inspired by the Hall–Heroult process, researchers tried to apply the “sand-to-silicon” electrolysis process to industrial production in 1957 [32]. Cryolite (Na_3_AlF_6_) was chosen as the molten salt because of its accessibility and success in aluminum electrolysis [33–35]. The SiO_2_/Na_3_AlF_6_ system was studied in some detail by Monnier’s group. Their experimental data showed that 99.9% to 99.99% purity silicon could be obtained at laboratory and pilot scales [36]. However, the project failed to achieve the goal of commercial development due to the precipitation of poorly conductive silicon in solid form on the electrode surface. The electrolytic deposition rate slows as the electrolysis advances, while the tank voltage increases, hindering continuous silicon production [37]. Huggins and Elwell [38] analyzed the limitations of the electrolytic silicon deposition process. They derived a mathematical approximation for the limited deposition rate [38], meaning that the maximum electrodeposition rate is predictable. Almost all systems used for solid electrodeposition had difficulties performing in a stable way and impurity-free at deposition rates above a few tens of micrometers per hour. However, this limitation does not affect liquid-phase electrodeposition, so the Hall–Heroult process for the commercial production of aluminum is feasible precisely because electrolysis is performed at temperatures above the melting point of aluminum. To solve the problem of the solid-phase cathode product after electrolysis, researchers at Stanford University developed (1978 to 1981) a feasible electrolytic “Hall–Heroult” process for the electrodeposition of silicon above its melting point [29]. Mattei et al. [39] conducted a detailed investigation. Silicon close to 4 N purity was obtained by electrolysis of barium silicate melt. The impurities in the product, including Ti, Al, and Fe, mainly originated from the SiO_2_ source. However, the relatively high melting point of silicon (1,414 °C) inevitably raised the quality requirement of the equipment and the energy consumption.

Although the “Hall–Heroult” process for silicon did not achieve large-scale application and production, we benefited from gaining knowledge of these studies. Many technologies and concepts developed for aluminum electrolysis are still widely employed today. For example, molten salt electrolytes containing calcium oxide and calcium chloride have been shown to facilitate silica dissolution at temperatures (850 °C) far below the Si melting point, with electrodeposited polysilicon films of sufficient purity for applications in functional PV devices, as discussed in detail in the “Design of electrodeposited crystalline Si films: Photoactive layers and *p*–*n* junction”.

### Electrodeposition of silicon films from fluorosilicate/fluoride-based melts

Besides the electrowinning of liquid silicon at ultra-high temperatures, as discussed above, another pioneering technique proposed by Stanford University’s Materials Research Center in the early 1970s was the direct deposition of silicon films in fluorosilicate/fluoride solutes [40], which simplified the tedious process of silicon wafer fabrication. As a by-product of the fertilizer industry, fluorosilicate is a rich and inexpensive silicon precursor for the electrochemical extraction of silicon. There are many types of electrolytes to choose from in the fluorosilicate/fluoride system, among which the most extensively studied are eutectic molten salt mixtures such as LiF–KF, NaF–KF, and LiF–NaF–KF, as they can obtain high-quality silicon films at temperatures well below the melting point of silicon [41,42]. Boen and colleagues [43,44] systematically investigated the effect of cathode substrates on silicon films prepared by electrodeposition in fluorosilicate/fluoride systems. The results show that silver was a suitable substrate for depositing continuous and dense silicon layers due to the ease of silicon nucleation and growing crystalline silicon on its surface. However, considering the high price of silver, the research focus was shifted toward inexpensive substrates such as graphite.

Several mechanisms and theories were suggested regarding silicon electrodeposition in fluorosilicate/fluoride systems. It was proposed that the reduction process is a quasi-reversible reduction of Si^4+^ to Si. The charge transfer process is the rate-controlling step during the deposition [45,46]. However, Boen and Bouteillon [43] proposed a 2-step electroreduction mechanism (Si^4+^→Si^2+^→Si) for SiF_6_^2−^ in the ternary eutectic melt Li–NaF–KF. Bieber et al. [47] suggested that the electrodeposition process of K_2_SiF_6_ in the NaF–KF molten salt involves transient nucleation and diffusion-controlled growth. Cai et al. [48] investigated the electrochemical reduction and nucleation of SiF_6_^2−^ in LiF–NaF–KF molten salt and concluded a one-step 4-electron mechanism. As shown above, the mechanism concerning the electrodeposition of silicon in fluorosilicate/fluoride-based systems is still controversial, and further investigation is necessary.

The silicon films obtained in all the early related works failed to exhibit PV effects due to the high corrosiveness of fluoride-based molten salts, which led to difficulties in impurity control. Electrodeposition of K_2_SiF_6_ dissolved in chloride-based molten salts (e.g., NaCl–KCl and LiCl–KCl) has also been attempted. However, the solubility of fluorosilicate is very low throughout the electrodeposition process, and the deposited silicon films were discontinuous and poorly smooth [26]. In the last decade, many studies have confirmed that water-soluble KCl–KF as a molten electrolyte can be used to deposit higher-quality silicon films [49–52]. The advantages are that KF–KCl salts can dissolve K_2_SiF_6_ well at high temperatures, and the cooled and cured KF–KCl salts have high solubility in water, making it easy to remove solid impurities adhering to Si deposits by aqueous washing.

**Fig. 3.**
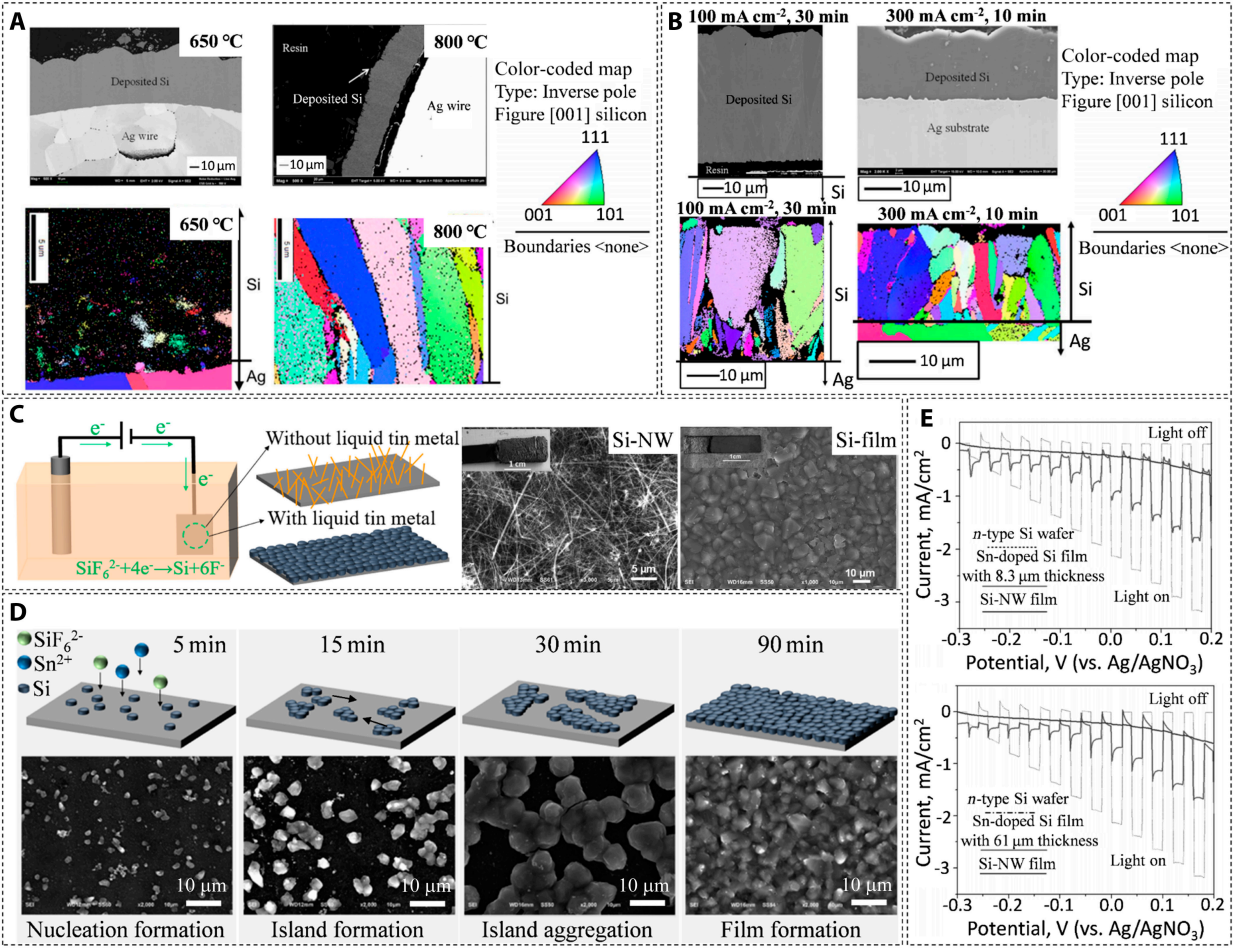
(A and B) Cross-sectional field emission scanning electron (FE-SEM) micrographs and crystal grain maps from electron backscatter diffraction (EBSD) analysis of the Si deposited under different electrolytic conditions in molten KF–KCl–K_2_SiF_6_ (2.0 mol%). (A) Silicon deposited on silver wire electrodes—current density of 155 mA cm^−2^ at 650 °C (left) and 800 °C (right). (B) Silicon deposited on Ag plate electrodes at 100 mA cm^−2^ for 30 min (left) and 300 mA cm^−2^ for 10 min (right). Reproduced from [51] with permission from IOP Publishing. (C) Schematic representation of the silicon products showing different morphologies on graphite sheet cathode in K_2_SiF_6_–KCl–KF system and their corresponding SEM micrographs. (D) Schematic representation of the growth mechanism of tin-doped silicon film via electrodeposition and its corresponding SEM micrographs. (E) Photoelectrochemical characterization of the electrodeposited silicon nanowires, silicon films, and *n*-type commercial silicon wafers. Reproduced from [53] with permission from Wiley-VCH.

Nohira’s group systematically investigated and verified that high-quality silicon films could be obtained by electrodeposition from K_2_SiF_6_ in KF–KCl molten at 650 °C, revealing that the optimal electrolytic conditions were as follows: K_2_SiF_6_ (2 to 3.5 mol%) and current density (50 to 200 mA cm^−2^) [49]. The authors also pointed out the relationship between the conditions of the electrolysis and the morphology of deposited silicon, i.e., the morphology of the silicon films changed from compact and smooth to nodular or coral-like with increasing current density and K_2_SiF_6_ concentration. Subsequently, the effect of temperature and current density was further investigated and discussed [51]. Figure 3A depicts the characteristics of the silicon deposits at 650 and 800 °C. Since the crystallization rate at 650 °C is slower than the deposition rate, only fine silicon grains are deposited. In contrast, larger columnar silicon crystals can be grown at a higher temperature (800 °C). The size of the silicon grains deposited at 100 and 300 mA cm^−1^ are 15 × 30 μm and 5 × 15 μm, respectively (Fig. 3B). This indicates that the deposited silicon grains will become smaller as the current density increases. Recent studies have shown that silicon films prepared in the KF–KCl–K_2_SiF_6_ system reach a 4 N purity level and exhibit *n*-type semiconductor characteristics [52–55]. Interestingly, high-purity SiCl_4_ can also be used as a silicon source to produce the same quality silicon films, which exhibit typical *p*-type semiconductor characteristics. Since gaseous SiCl_4_ dissolved in KF–KCl forms SiF_6_^2−^ (or SiF*_x_*Cl*_y_*^(*x*+*y*−4)−^) anions, the system is thus identical to the silicon electrodeposition in the molten KF–KCl–K_2_SiF_6_ system, as shown in Eqs. 4 and 5. However, the formation and tuning of the *p-*type and *n*-type silicon films still need further investigation.

$${\mathrm{SiCl}}_{4}\left(g\right)+6F^{–}\to {\mathrm{SiF}}_{6}^{2–}+4{\mathrm{Cl}}^{–}$$ (4)

$${\mathrm{SiF}}_{6}^{2–}+4e^{–}\to \mathrm{Si}+6F^{–}$$ (5)

Given the advantages of the KCl–KF–K_2_SiF_6_ system, Peng et al. [53] conducted a study on the direct electrodeposition of photoactive silicon films; the products were obtained in the form of silicon films and silicon nanowires, which were doped or undoped with Sn^4+^, due to the presence of liquid tin (Fig. 3C). Liquid tin has a crucial role as a medium to promote all the growth stages of dense continuous silicon films: island formation/aggregation and film formation, as shown in Fig. 3D. In addition, the tin-doped silicon film exhibited *n*-type semiconductor behavior, generating photocurrents. The results were acceptable, achieving ~38 to 44% of commercial *n*-type silicon wafers (Fig. 3E). However, the authors observed dark currents, implying the presence of undesired pinholes and cracks in the silicon film, which may be due to the aggressiveness of KF in the solute. Although the quality of the deposited silicon in KCl–KF has been improved considerably, the corrosive effects of fluoride-based molten salt remain challenging to overcome. Therefore, Laptev et al. [54] tried to replace part of the KF and KCl with a high concentration of KI to reduce the solute’s aggressiveness further. Glassy carbon and tungsten substrates were used to obtain silicon films with good adhesion. The experimental parameters were as follows: KF–KCl (2:1) −75 mol% KI containing 0.075 or 0.5 mol% K_2_SiF_6_ at a temperature of 725 °C. In addition, the authors also confirmed that the solute could be used to obtain *n*-type silicon films by adding AlF_3_ as a dopant [55], but further studies on the purity and optical properties of the films are necessary.

As discussed above, dense and smooth silicon films with *p*-type or *n*-type semiconductor properties were obtained in KF–KCl or KF–KCl–KI molten salts using fluorosilicates or SiCl_4_ as the silicon source. Although the control of *p*/*n* junction semiconductors was not achieved then, the possibility of applying the proposed strategy to produce PV silicon films has been elucidated.

### Electrodeposition of amorphous Si from organics

The electrodeposition of amorphous silicon also makes excellent sense for solar cells. Since amorphous silicon can be deposited at temperatures close to ambient, the energy cost of electrodepositing amorphous silicon in organic solvents (electrolytes) is extremely low compared to high-temperature molten salts [56,57]. The electrodeposition process should be performed under an inert atmosphere because the precursors [e.g., SiX_4_ or SiHX_3_ (X = Cl, Br)] tend to react with moisture in the air to form oxides through reaction (6). The first report on the electrodeposition of amorphous silicon was conducted by Agrawal and Austin [58], who prepared 1- to 3-μm-thick Si films from organic solvents on various substrates (such as platinum, titanium, and silicon wafers) at temperatures ranging from 35 to 145 °C using SiX_4_ and SiHX_3_ (X = Cl, Br) as precursors. However, the obtained silicon films contained ~3% SiO_2_, traces of metallic impurities, and some Si–H and Si–H_2_ entities. The ethyl orthosilicate electrodeposition in acetic acid, propylene carbonate, and 1-chloropropane solvents has also been investigated. Although electrodeposition under these conditions appears promising, these deposits produce large amounts of SiO_2_ upon exposure of the inclusions to air.

$${\mathrm{SiH}}_{4}+2H_{2}O\to {\mathrm{SiOX}}_{3}+\mathrm{HX}\;\mathrm{to}\;{\mathrm{SiO}}_{2}+4\mathrm{HX}$$ (6)

Gobet and Tannenberger [59] utilized SiHCl_3_, SiCl_4_, or SiBr_4_ as precursors and tetrahydrofuran (THF) as the solvent to deposit silicon films on Pt, Au, Ni, Cu, glassy carbon, and In-Sn oxide (ITO) substrates. The resulting thickness of the films was only 0.25 μm. However, the film also contained impurities such as carbon (~8 at.%), oxygen (~8 at.%), and chlorine (1.5 at.%). Nicholson [60] reported that silicon was electrodeposited on *n*-type silicon and titanium substrates using SiCl_4_ and SiBr_4_ as precursors in propylene carbonate and THF as solvents. The deposited silicon showed honeycombed morphology and contained some impurities (similar to the previous case): carbon, hydrogen, oxygen, and chlorine. Interestingly, the obtained Si showed photo-responsiveness, which may be generated by pure and non-oxidized deposits close to the substrate. In addition, adding a low concentration of AlCl_3_ in this system as a *p*-type dopant induces the formation of *p*–*n* junctions on silicon wafers. Nishimura and Fukunaka [61] electrodeposited silicon film up to a 50-μm thickness from SiCl_4_ in propylene carbonate, but with very high levels of other species and immediate oxidation upon contact with air. Munisamy and Bard [62] reported the electrodeposition of silicon on Ni, Ag, and glassy carbon from acetonitrile and THF containing the SiCl_4_ and SiHCl_3_ precursors. Similarly, the deposits were immediately oxidized as soon as they were exposed to air, and even the least exposed fraction contained significant amounts of carbon and oxygen. The authors pointed out that annealing the deposited silicon at 350 to 850 °C effectively reduces the C, N, and H concentrations, thus raising the quality of silicon. Besides, the annealing process induces silicon crystallization and results in weak p-type photoactivity.

In principle, amorphous silicon electrodeposition from organic solvents is an attractive strategy, which is very economical and convenient compared to a high-temperature deposition. However, the poor quality of the silicon films due to their extreme susceptibility to oxidation and absorption of other species from organic solvents resulted in partially abandoning this approach. No solution to the problem of silicon oxidation has been found, at least so far. Therefore, further work should focus on the appropriate antioxidant strategies and control the content of other species in silicon deposits.

## State-of-the-Art Silicon Electrochemical Extraction Strategies and Current Advances

### Electrochemical methods for “SiO_2_ to Si” in molten chloride salt

The cathodic electrochemical deoxidation (reduction) of metal oxides, called the FFC Cambridge process, has attracted increasing attention from scholars in various countries since its discovery by Chen et al. [63]. The principle of this method is to apply a voltage to the solid oxide as a cathode at a temperature below the melting point of the metal and the decomposition voltage of the molten salt electrolyte (e.g., CaCl_2_-based molten salt), whereby the oxygen is removed by ionization, resulting in a metallic material. Initially, this process was only used to purify metals such as nickel and titanium [64]. Still, as the process was refined and extended, it was applied to silicon extraction.

**Fig. 4.**
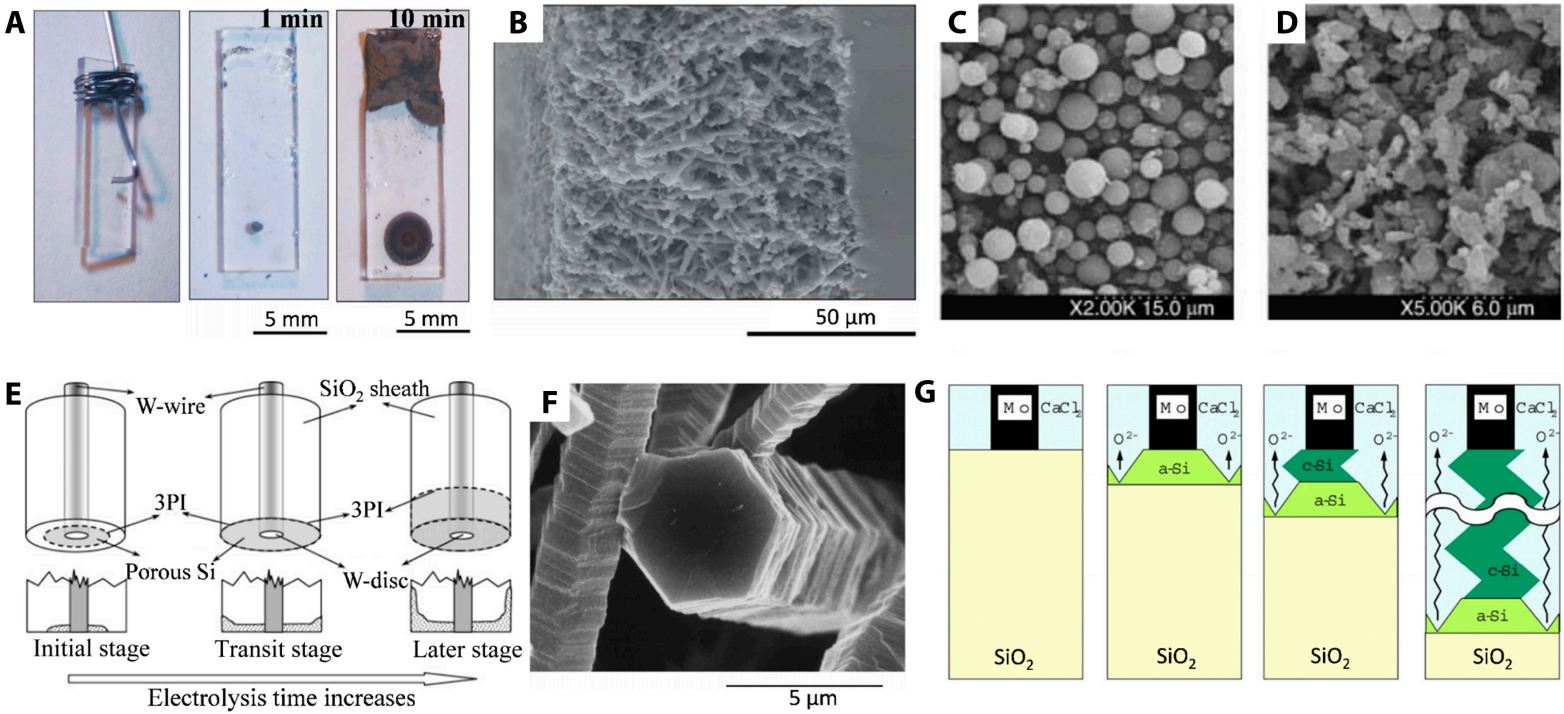
(A) Optical photographs of quartz electrode before and after electrolysis in molten CaCl_2_ at 850 °C. (B) SEM micrograph of quartz contact area after electrolysis. Reproduced from [24] with permission from Springer Nature. (C and D) SEM micrographs of micrometer-sized SiO_2_ powder before (C) and after (D) electrolysis. Reproduced from [65] with permission from Wiley-VCH. (E) Schematic illustrations of the 3PI reaction mechanism. Reproduced from [68] with permission from Wiley-VCH. (F) SEM micrographs of the Si columns obtained by electrochemical reduction of SiO_2_ in molten CaCl_2_ at 850 °C. (G) Schematic illustration of the formation mechanism of Si columns. Reproduced from [69] with permission from IOP Publishing.

In 2003, Nohira et al. [24] achieved a partial reduction of SiO_2_ by electrolysis of solid quartz in CaCl_2_ and LiCl–KCl–CaCl_2_ molten salts by the FFC Cambridge process. The photographs and SEM micrographs show that the reduced SiO_2_ is mainly in contact with the Mo wire (Fig. 4A and B). Immediately after, Jin et al. [65] obtained pure silicon powder by direct electrolytic reduction of SiO_2_ porous cathode pellets in molten CaCl_2_ at 850 °C. SiO_2_ particles with diameters of 2 to 7 μm are converted into pure silicon of 1 to 3 μm after electro-deoxidation (Fig. 4C and D). The porous pellets (0.5 mm thickness) required less than 4 h to achieve complete reduction with an energy of 13 kWh kg_Si_^−1^. Due to the chemical inertness of silicon, the electrolysis product can be washed after removal from the salt, and the residual impurity metals and oxides therein can be removed using dilute acids. This discovery provided a new definition of silicon production, attracting strong interest from the international academic community and the energy industry.

#### The SS mechanism and its process

This mechanism is assumed to be identical to the FFC Cambridge process. This involves directly reducing solid oxides to metals/alloys by electrochemical deoxidation in molten chlorides [66]. However, unlike electrolytic TiO_2_, SiO_2_ is an insulator. It has no low-valent oxide phase, which is directly reduced to crystalline silicon by gaining 4 electrons. Solid silica insulators’ direct electrochemical reduction mechanism starts when the reaction occurs in the conductive collector/silica/molten salt contact region. When a sufficient cathodic overpotential is applied to the silica precursor, electrons are transferred from the conducting collector through the collector/oxide interface into the oxide phase. Simultaneously, oxide anions in the silica migrate through the oxide/electrolyte interface into the molten salt electrolyte. Solid silica is reduced to conductive monolithic silicon, causing an increase in molar volume. The product forms a porous layer, and the molten salt enters through the pores, forming a new Si/silica/molten salt 3-phase interline (3PI) reaction area. The continuous expansion of the 3PIs at the surface and in the bulk phase results in the complete reduction of silica. Chen and colleagues [67,68] modeled the corresponding 3PI boundary model by the SiO_2_-sheathed electrode method, as shown in Fig. 4E. Thus, this is also called the 3PI reaction mechanism.

$${\mathrm{SiO}}_{2}+4e^{–}\to \mathrm{Si}+2O^{2–}$$ (7)

The morphology change and crystallization for Si occurred during the electrolytic reduction of SiO_2_ [69]. The formed Si exhibited a typical hexagonal prismatic structure with numerous stacking faults along the <111> direction (Fig. 4F). During the electrolysis, SiO_2_ is first reduced to amorphous silicon with a fluffy structure, which immediately transformed into crystalline silicon due to the formation of a new SiO_2_/Si/CaCl_2_ 3PI (Fig. 4G). Notably, the generation of amorphous silicon from SiO_2_ is the key step of the process. The O^2−^ diffusion in the obtained crystalline silicon vacancies controls the electrochemical reduction rate.

#### From SS to SLS: The crucial role of CaO and O^2−^

**Fig. 5.**
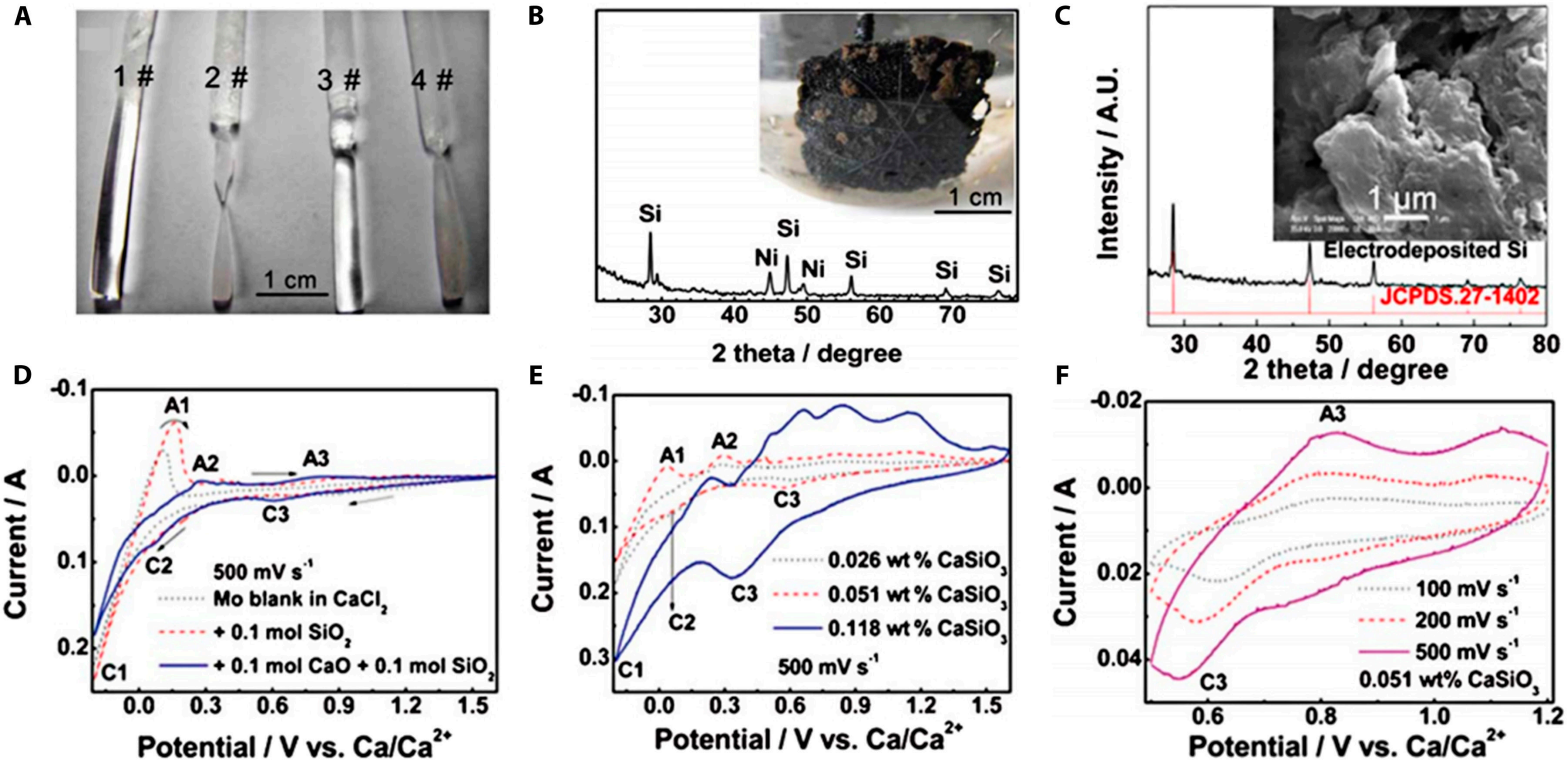
(A) Photographs of quartz bars after immersion in molten CaCl_2_ (1#) and CaO–CaCl_2_ (2#, 3#, and 4#). (B) Photograph of silicon-deposited (on the outer surface) nickel foil and its corresponding x-ray diffraction (XRD) pattern. (C) XRD patterns and SEM micrographs of Si powders electrodeposited on Ni substrates. (D) CVs of the Mo electrode in molten CaCl_2_ (dotted), CaCl_2_–SiO_2_ (dashed), and CaCl_2_–SiO_2_–CaO (solid) at 850 °C. (E) CVs of Mo electrode in molten CaCl_2_ with different CaSiO_3_ content at 850 °C. (F) CVs of Mo electrode in CaCl_2_ melt containing 0.051 wt.% CaSiO_3_ at different scan rates. Reproduced from [71] with permission from the Royal Society of Chemistry.

Initially, the SLS mechanism (i.e., the dissolution–electrodeposition mechanism of silicon, which we replace with “SLS” in this paper) was not favored because the solubility of SiO_2_ in molten CaCl_2_ was too low. However, in the actual electrolytic reduction of SiO_2_, silicates (denoted as Ca*_y_*Si*_x_*O_2*x*+*y*_, e.g.*,* CaSiO_3_, Ca_2_SiO_4_, and Ca_3_Si_2_O_7_) as intermediate products changed this situation [70–73]. CaCl_2_ will always undergo a hydrolysis reaction, and its solute will contain CaO independent from any pretreatment processes applied. As shown in Eqs. 8 and 9, CaO will interact with SiO_2_ to form silicate anions (expressed as SiO*_y_*^*n*−^, including SiO_3_^2−^ and SiO_4_^4−^) because the Gibbs free energy change for this reaction at 850 °C is −139.76 kJ mol^−1^. The addition of 4.8 mol% CaO to CaCl_2_ by Kongstein et al*.* [74] was found to be beneficial for the dissolution of SiO_2_. Until 2012, Xiao et al*.* [71] systematically investigated the mass loss of SiO_2_ (quartz rods) immersed in molten CaCl_2_ containing different amounts of CaO (0, 2, 3, and 5 mol% corresponding to 1#, 2#, 3#, and 4# in Fig. 5A) for 5 h. The solubility of SiO_2_ in molten CaCl_2_ increased with the concentration of CaO (Fig. 5A). Moreover, the authors observed a layer of silicon deposited on the outer surface of the nickel foam substrate during electrolysis of SiO_2_ in CaCl_2_ (containing 2 mol% CaO). Interestingly, the nickel foam substrate was not in direct contact with SiO_2_ (Fig. 5B). Pure silicon can be obtained on the surface of the nickel substrate when long electrolysis is carried out (Fig. 5C). This anomalous deposition of silicon provides evidence for the existence of an SLS mechanism.

The dissolution of SiO_2_ with the assistance of CaO was also confirmed by cyclic voltammetry (CV). As shown in Fig. 5D, with the addition of only 0.1 mol of SiO_2_, there are no prominent redox peaks in the CV curve (dashed line), except for Ca formation (C1/A1). With the addition of 0.1 mol of CaO, 2 pairs of redox peaks (C2/A2 and C3/A3) appear in the corresponding CV (solid line), which are attributed to the redox reaction of the dissolved silicate. The CV of the Mo electrode in molten CaCl_2_ (with different CaSiO_3_ contents) is shown in Fig. 5E. The C3 peak with an initial potential of 0.75 V indicates the formation of electrodeposited silicon, which is the same as the result of the CaCl_2_–CaO–SiO_2_ system (Fig. 5D). Furthermore, the C3 peak intensifies in the CV at faster sweep rates, demonstrating the mechanism of electrodeposition of silicon in the melt. The current increases with increasing sweep rate (Fig. 5F), which is characteristic of the electrochemistry of dissolved species.

Furthermore, during the molten salt electrolysis of solid SiO_2_, the slow diffusion kinetics of O^2−^ results in a high concentration at the electrochemical interface. This induces the rapid combination of O^2−^ with solid SiO_2_ near the cathode to form silicates. When the soluble silicate concentration reaches a specific value, it is sufficient to trigger the silica deposition Eqs. 10 and 11. Thus, silicate is bound to be present on the cathode regardless of whether CaO is added to the CaCl_2_ electrolyte. The SLS mechanism depends on the concentration of O^2−^ near the solid SiO_2_ cathode, which is also an important factor controlling the process. The O^2−^ concentration can be adjusted by various parameters, including electrolytic potential, temperature, and CaO concentration. The SLS mechanism in the electrolytic reduction of SiO_2_ drives the completion of electrolysis together with the mechanism of SS deoxygenation reaction.

$${x\mathrm{SiO}}_{2}+y\mathrm{CaO}\left({\mathrm{Ca}}^{2+},O^{2–}\right)\to {\mathrm{Ca}}_{y}{\mathrm{Si}}_{x}O_{2x+y}\left({y\mathrm{Ca}}^{2+},{\mathrm{Si}}_{x}O_{2x+y}^{2y–}\right)$$ (8)

$${\mathrm{Ca}}_{y}{\mathrm{Si}}_{x}O_{2x+y}\to {y\mathrm{Ca}}^{2+}+{\mathrm{Si}}_{x}O_{\left(2x+y\right)}^{2y–}$$ (9)

$${x\mathrm{SiO}}_{2}+yO^{2–}\to {\mathrm{Si}}_{x}O_{\left(2x+y\right)}^{2y–}$$ (10)

$${\mathrm{Si}}_{x}O_{\left(2x+y\right)}^{2y–}+4{xe}^{–}\to x\mathrm{Si}+\left(2x+y\right)O^{2–}$$ (11)

#### Controllable design of nanostructured silicon: Nanowire and nanotube

Nanostructured silicon materials provide unprecedented opportunities for a wide range of applications in sensors [75], optics [76], nanoelectronics [77], biocatalysis [78], and energy storage [27,79]. Nanostructured silicon materials (e.g., nanowires and nanotubes) exhibit significantly enhanced performance as Li-ion battery anodes due to the efficient mitigation of the volume expansion of silicon, as well as shorter diffusion channels for Li^+^ [12].

**Fig. 6.**
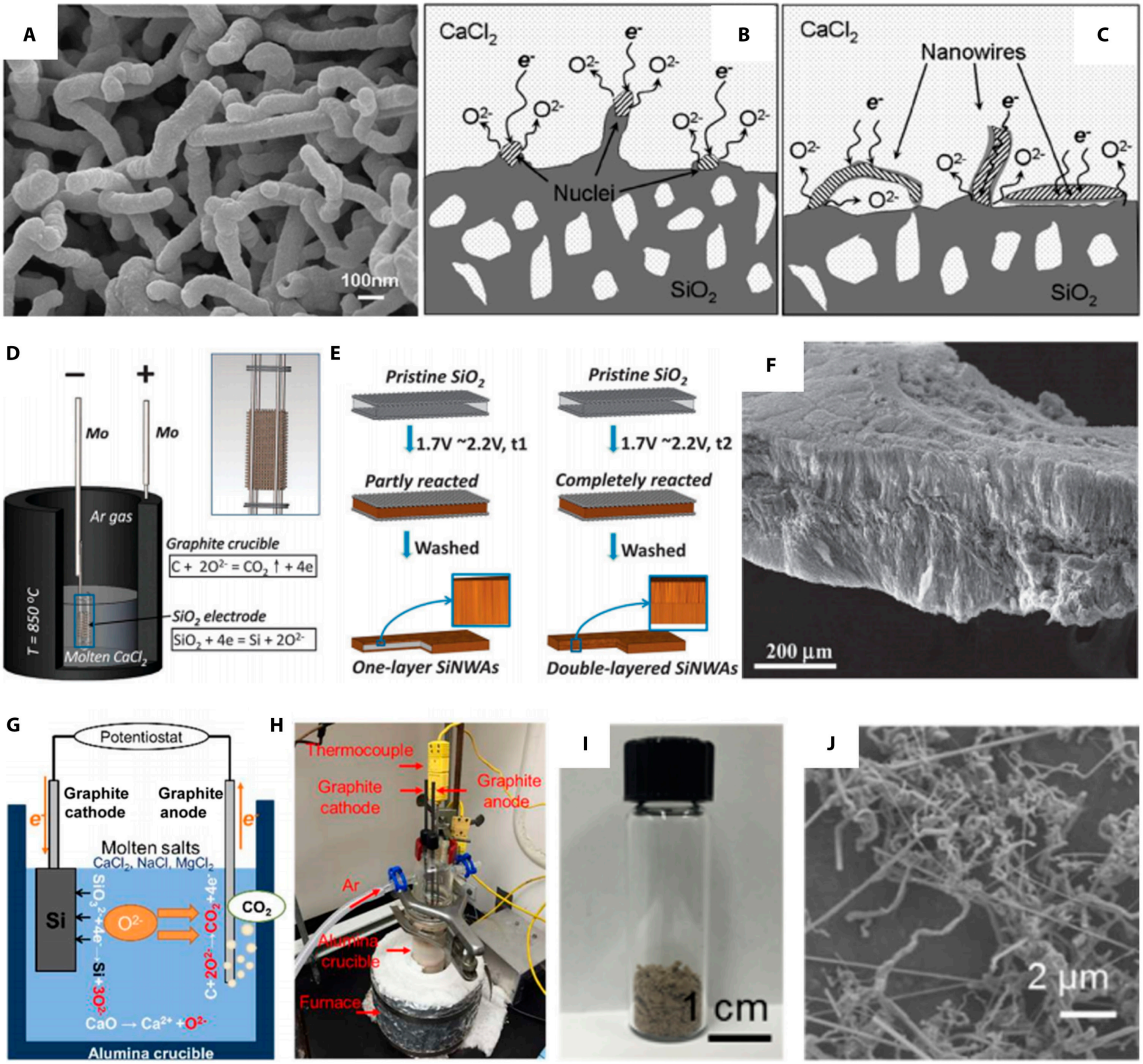
(A) Si-NWs obtained by electrolysis in molten CaCl_2_ at 900 °C at −1.20 V. (B and C) Schematic diagram of (B) Si-NW nucleation and (C) growth mechanism during the electrochemical reduction process. Reproduced from [80] with permission from The Royal Society of Chemistry. (D) Schematic illustration of the electrolysis system for the preparation of silicon. (E) Schematic diagram of the synthesis process of Si-NW arrays. (F) SEM micrographs of the obtained double-layered Si-NW arrays. Reproduced from [82] with permission from The Royal Society of Chemistry. (G) Schematic illustration of the electrolytic cell and (H) the equipment for the preparation of silicon in molten salts. (I and J) Photograph and SEM micrograph of Si-NWs. Reproduced from [84] with permission from Wiley-VCH.

Generally, the employment of nano-silica as starting material is considered a prerequisite for the electrolytic synthesis of nanostructured Si. Yang et al*.* [80] reported the formation of Si nanowires (Si-NWs) by electrolysis in a molten salt using porous nano-SiO_2_ powder as raw cathodic material (Fig. 6A). The authors proposed a corresponding growth mechanism (Fig. 6B and C). The silica near the metal electrode first undergoes deoxygenation and reduction to silicon. Since the latter is conductive at high temperatures, it will act as a nucleus to form 3PIs with unreduced silica nanoparticles and molten salt. As the reaction continues, the silicon nuclei grow in the direction of the 3PIs, resulting in the formation of nanowires. In the last decade, silicon nanowires with various characteristics have been developed by molten salt electrolysis. Bent and entangled high-purity silicon nanowires were produced by cathodic electro-deoxidation in molten CaCl_2_ at 850 °C using porous silicon particles as raw material in the potential range of 0.65 to 0.95 V (versus Ag/AgCl) [81]. Free-standing bilayer silicon nanowire arrays with diameters of 50 to 200 nm and thicknesses up to 100 μm can be prepared (Fig. 6D to F) by 2 nickel grids sandwiched by a quartz cathode in molten CaCl_2_ [82]. Alternatively, high-purity straight silicon nanowires can be obtained by adding catalysts to the precursors. For example, porous particles composed of metallic Ni (0.8 wt.%) and SiO_2_ can be electrochemically converted into silicon nanowires [83].

However, the electro-reduction of silica is mainly driven through the 3PI reaction mechanism, which results in very low silicon yields. To address this limitation, Dong et al. [84] used soluble CaSiO_3_ as the precursor to continuously produce silicon nanowires by electrodeposition (Fig. 6G and H). Unlike direct electrolysis of SiO_2_, CaSiO_3_ can be dissolved directly in CaCl_2_-based molten salts to generate Ca^2+^ and SiO_3_^2−^, while SiO_3_^2−^ is reduced to Si (8) at a certain potential. The rate-controlling step is the diffusion of O^2−^, which can be accelerated by adding CaO. Furthermore, the electrolysis temperature can be lowered to 650 °C using a ternary eutectic melt of CaCl_2_–MgCl_2_–NaCl (Fig. 6I and J). More significantly, it is demonstrated that glass waste or coal ash composed of SiO_2_, CaO, and Na_2_O can also be used as starting materials for this process, opening a new path for the sustainable production of silicon nanowires. It should be mentioned that a ton-scale pilot plant producing silicon nanowires by the electrolysis of silica in molten salt was put into function in China [85].

**Fig. 7.**
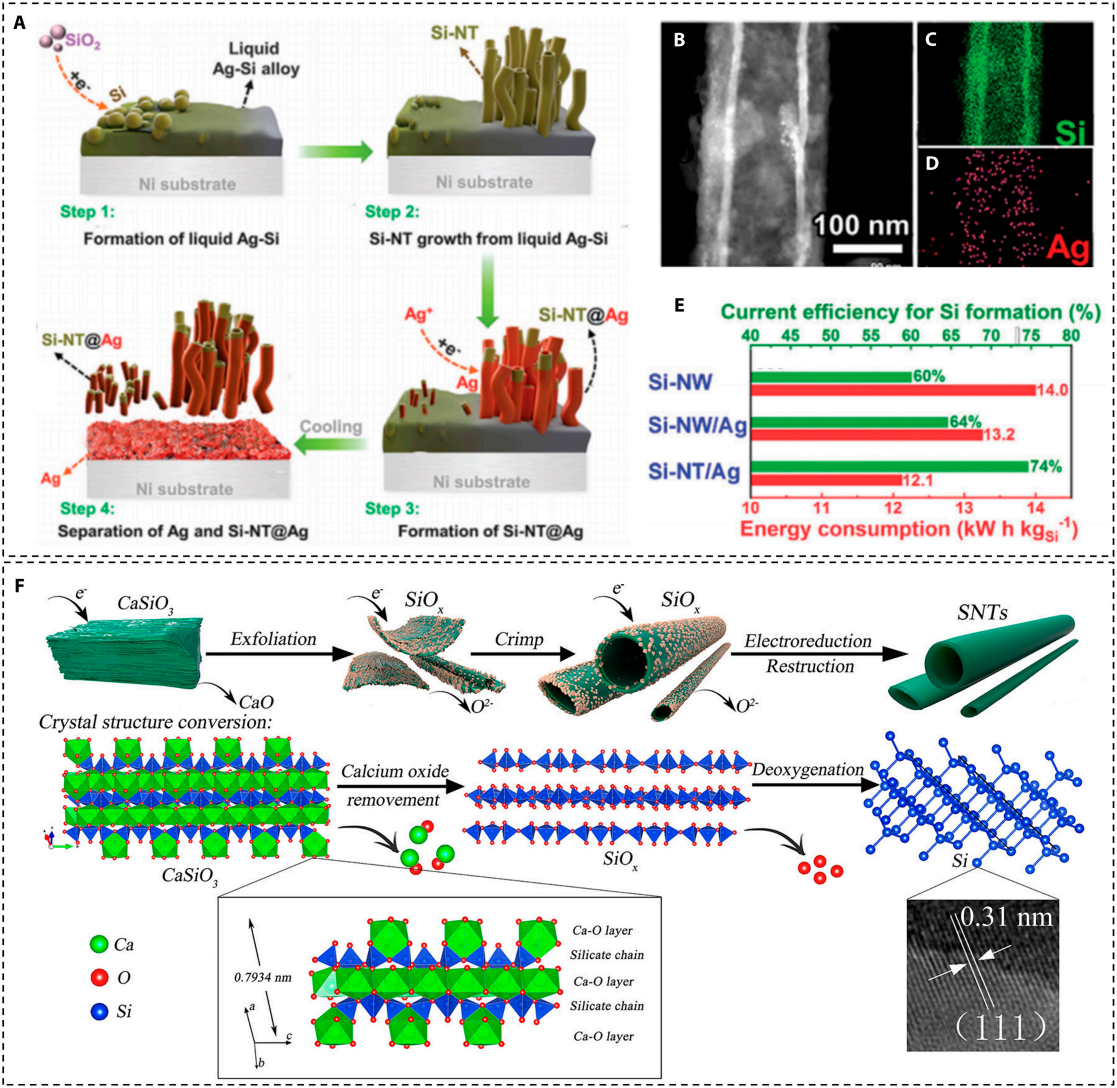
(A) The formation mechanism of Si-NTs and Si-NT@Ag using SiO_2_–AgCl mixtures as a precursor in NaCl–CaCl_2_ molten salts at 850 °C. (B to D) Transmission electron microscope (TEM) micrographs and EDS maps of Si-NTs. (E) Current efficiency and energy consumption in the synthesis of different samples. Reproduced from [86] with permission from Wiley-VCH. (F) Schematic illustration of direct electroreduction of solid CaSiO_3_ in CaCl_2_/NaCl molten salt to obtain Si-NTs. Reproduced from [87] with permission from the American Chemical Society.

Except for Si-NWs, silicon nanotube (Si-NT) synthesis by molten salt electrolysis has also been investigated and reported in recent years [86–90]. Weng et al. [86] achieved the electrochemical synthesis of Si-NTs and Si-NT@Ag on Ni substrates by co-electrolysis of SiO_2_ and AgCl in molten NaCl–CaCl_2_ at 850 °C. As shown in Fig. 7A, the whole Si-NT and Si-NT@Ag growth process can be divided into 4 steps. First, Ag and Si were sequentially deposited on the Ni substrate. When their concentration reaches a certain level, liquid Ag–Si alloy is formed at 850 °C. Subsequently, the continuous electroreduction and deposition resulted in the supersaturation of Si, which induced the formation of Si-NT via a liquid–solid mechanism (steps 1 and 2 in Fig. 7A). Interestingly, Si-NTs were covered with Ag (Si-NT@Ag, verified by energy dispersive spectrometer (EDS) after adding saturated silver chloride (step 3 in Fig. 7A). The Ag deposited appeared as a furry coating on Si-NTs (Fig. 7B to D). In addition, the authors pointed out that Si-NTs and Si-NT@Ag were automatically separated from Ag and impurities during the annealing process, thus ensuring the purity of the product (step 4 in Fig. 7A). Most importantly, this strategy ranks among the best in both current efficiency and energy consumption (Fig. 7E). This process has even higher efficiency than that of industrial production of metallurgical silicon, which is very promising. Wang and colleagues [87,88] directly selected layered CaSiO_3_ as the starting material, which accelerated the formation of Si-NTs (Fig. 7F). CaO is exfoliated from CaSiO_3_ during electrolysis to form SiO*_x_* (0 < *x* < 2) sheets. It should be noted that, unlike the typical silicon SLS mechanism proposed in previous reports, this Si-NT formation is a typical SS reduction process. Furthermore, the authors found that SiO_2_ particles can also be used as a starting material for Si-NT production, where SiO_2_ in the molten salt reacted with CaO to form CaSiO_3_ during the initial stage of electrolysis, followed by the same reduction mechanism as described above.

The design of Si surface nanostructures by electrochemical deoxidation in molten salts is also relevant. Of particular interest at present is the surface-engineered modification of silicon layers. For example, black silicon is a new electronic material that acts like a light-absorbing sponge that captures almost all visible and infrared light, so it can significantly improve PV conversion efficiency [91–94].

**Fig. 8.**
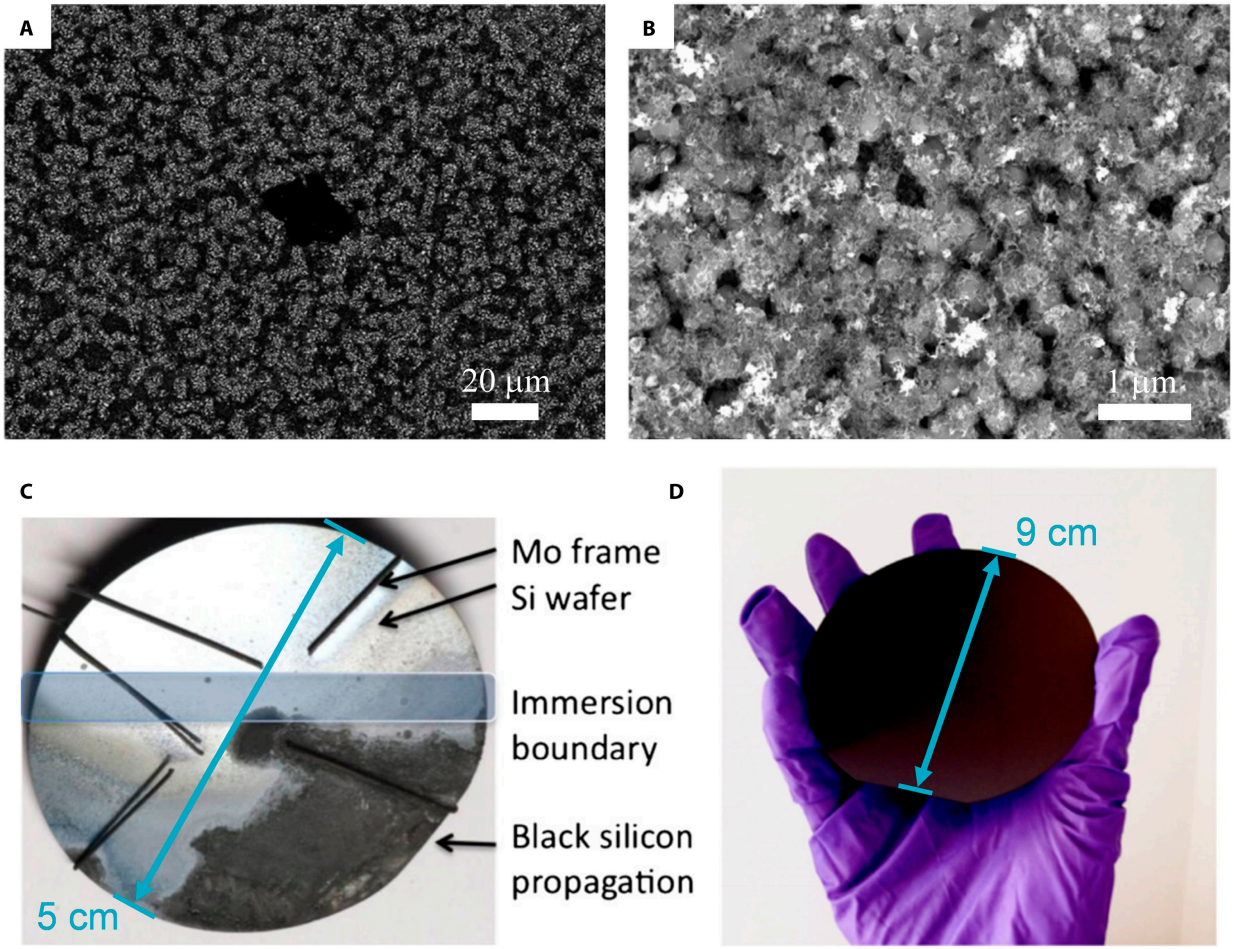
(A) Typical SEM micrograph of SiO_2_ thin layer after deoxidation. Reproduced from [95] with permission from Elsevier. (B) The porous spherical texture of SiO_2_ thin layer with a thickness of 2 μm after electrolysis. (C) Photograph of black silicon (black part) after being reduced on the wafer surface. Reproduced from [96] with permission from Elsevier. (D) Photograph of complete black silicon obtained by molten salt electro-deoxidation. Reproduced from [97] with permission from Elsevier.

The team of Fray reported a detailed investigation of the electrochemical production of black silicon in molten salt. In 2010 [95], fine nanoscale silicon with specific surface texture thin layers was obtained after electro-deoxidation in molten CaCl_2_. The starting material was *p*-type silicon wafers with a thermally oxidized surface (Fig. 8A). The authors pointed out that even silicon oxide layers with a thickness of only a few tens of nanometers can be converted to submicrometer-sized spherical particles under prolonged electrolytic conditions. Thus, shortening the electrolysis time is beneficial for generating superfine nanostructures on the surface. Subsequently, 2-μm-thick SiO_2_ film on the surface of *p*-type silicon underwent further electro-deoxidation in molten CaCl_2_ at 850 °C [96]. The results show that the surface of the electrolyzed SiO_2_ film is a porous lattice structure composed of nano-nuclei and nanofibers, as shown in Fig. 8B. The reduction of the oxide layer on the wafer surface propagates along the 3PIs, and the reduced region (black) is the obtained black silicon (Fig. 8C). Notably, the product shows significant visible light absorption, with a measured reflectance of ~8 to 11%. To further optimize the light absorption efficiency of the porous black silicon layer, TiO_2_ was deposited on the black silicon surface prepared by electroreduction in molten salt to obtain “an extremely black” surface with reflectivity as low as 0.1% (Fig. 8D) [97]. There is no doubt that these findings demonstrate the potential of electrochemical deoxidation for the industrial production of black silicon. However, an industrial-scale process is still awaited.

#### Design of electrodeposited crystalline Si films: Photoactive layers and *p*–*n* junction

As discussed in the “Electrodeposition of silicon films from fluorosilicate/fluoride-based melts” section, molten salt electrodeposition of silicon films provides a new opportunity to replace conventional silicon wafer production. Unlike the intensely aggressive fluoride, CaCl_2_, a low-cost, water-soluble, and eco-friendly salt, has been suggested as a promising electrolyte for depositing silicon film. Furthermore, it is known that silicates are generated in molten CaCl_2_, which can be reduced and deposited in the form of solid silicon at the cathode. Hence, producing high-quality Si films via electrodeposition in the CaCl_2_–SiO_2_ system is feasible.

In recent years, Bard’s group has conducted intensive and systematic research in this area. Cho et al. [98] attempted the direct electrodeposition of Si films in the CaCl_2_–SiO_2_ system. A relatively pure discontinuous crystalline Si film was obtained on Mo foil at 850 °C by applying a constant current. The raw material was 5- to 15-nm-sized SiO_2_ nanoparticles. However, no obvious photocurrent was observed. In general, the formation of silicon films is intimately related to the selected cathode substrate because they form by depositing nuclei on the substrate surface, forming continuous silicon particles and growing consistently. The surface of Mo is unfavorable for the deposition and growth of silicon nuclei. Therefore, the silver foil was further utilized to deposit photoactive silicon films from the CaCl_2_–SiO_2_ system [99]. The deposition of silicon on silver substrates is believed to occur via the formation of liquid Ag–Si alloy, followed by the precipitation of silicon after supersaturation in the liquid alloy and continued growth. The obtained silicon demonstrates high purity (>99.9%) as the impurities can be spontaneously separated during the precipitation of silicon from the supersaturated liquid. The obtained silicon exhibited a clear photoresponse, approaching half the maximum photocurrent of pure silicon wafers. However, the high price makes it difficult for a silver substrate to become more competitive in the market. The relatively inexpensive graphite has also been shown to be a suitable substrate for silicon as early as studies of fluoride electrodeposition. Zhao et al. [100] first reported that dense, continuous *p*-type silicon films were deposited on a graphite substrate in molten CaCl_2_ containing 0.3 mol% nano-SiO_2_ at a current density of 6 mA cm^−2^ for 1 h. The electrodeposited thin silicon films on graphite showed higher photocurrent than those obtained on silver.

**Fig. 9.**
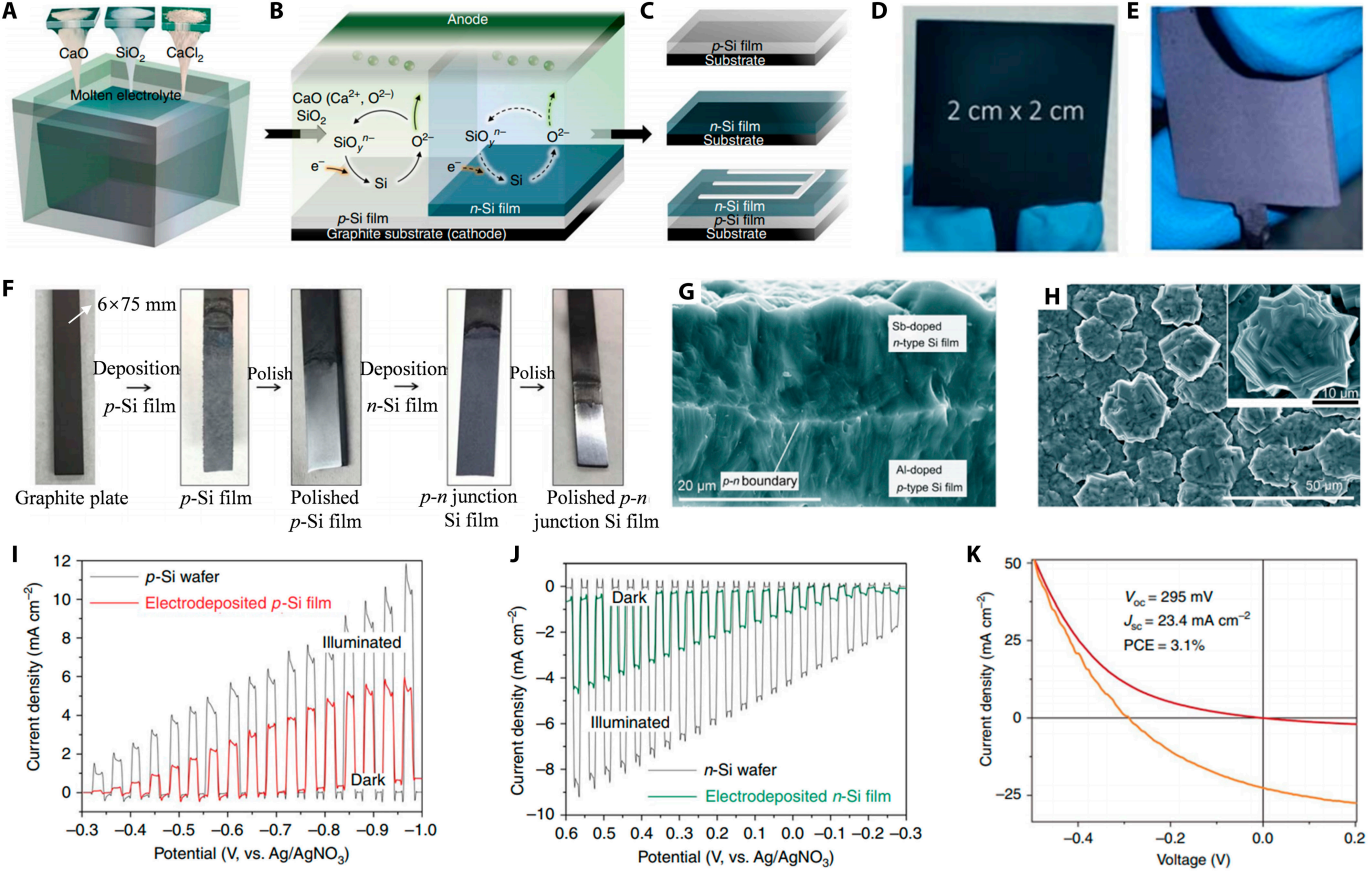
(A to C) Schematic representation of the electrodeposition process for crystalline silicon film production. (D and E) Photographs of the graphite substrate before and after crystalline silicon film deposition. (F) Typical photograph showing a 2-step electrodeposition process for fabricating *p*–*n* junction silicon films. Reproduced from [73] with permission from Springer Nature. (G and H) SEM micrographs of the cross-section and surface of a silicon *p*–*n* junction. Reproduced from [101] with permission from the American Chemical Society. (I and J) Photocurrent density–potential characteristics of the *p*-type/*n*-type silicon film and a commercial *p*-type/*n*-type silicon wafer in darkness and under illumination at 100 mW cm^−2^ with a scan rate of 10 mV s^−1^. (K) Current–voltage characteristics of the *n*-type silicon layer on a *p*-type single-crystalline silicon wafer under dark and 100 mW cm^−2^ illuminations. Reproduced from [73] with permission from Springer Nature.

It is well known that the low solubility of SiO_2_ nanoparticles in molten CaCl_2_ limits the net mass transport. Aiming to break this limitation, Bard and colleagues [72,73,101] demonstrated the study of continuous electrodeposition of photoactive Si films in the CaCl_2_–CaO–SiO_2_ system. As shown in Fig. 9A and B, during the electrodeposition process, O^2−^ ionized from CaO reacts with nano-SiO_2_ to form silicate ions. CaO, as an intermediate medium, facilitates the continuous ionization of SiO_2_ to form silicate ions, which are reduced to silicon crystallites on the graphite surface. To achieve high-purity silicon, periodic pre-electrolysis of molten CaCl_2_–CaO–SiO_2_ is necessary. The impurities in the deposited silicon film should be strictly controlled to be lower than the allowable values. Since the electrodeposition process is performed at a low current and the reactor is strictly protected by high-purity argon gas, any possible CO or CO_2_ emissions from the graphite anode are immediately removed. It should be pointed out that electrodeposition parameters (current density, time, etc.) are essential to improve the efficiency of the electrolytic process and to fabricate high-quality silicon films. Generally, the type and concentration of dopants can be directly controlled by adjusting the sources in the molten salt. *P*-type, *n*-type, and *p*–*n* junction silicon films were electrodeposited on a graphite substrate (Fig. 9C to F). Silicon *p*–*n* junction films were produced directly from SiO_2_–CaO–CaCl_2_ systems by a 2-step electrodeposition process, i.e., the *p*-type Si film was first electrodeposited on a graphite substrate with the aluminum dopant originating from the used quartz crucible or from the addition of additional Al_2_O_3_. Subsequently, the prepared *p*-type silicon film was polished and used as a substrate for the electrodeposition of *n*-type silicon film using Sb_2_O_3_ or Ca_3_(PO_4_)_2_ as the dopant sources. The fabricated dense *p*–*n* junction silicon film shows typical hexagonal crystalline particles (Fig. 9G and H) and a purity of 99.9998% (close to 6 N, solar grade), which is the highest purity ever reported in silicon electrodeposition research. The photocurrent density was approximately 40% to 50% of commercial silicon wafers, with a maximum power conversion of 3.1% as a solar cell (Fig. 9I to K).

As discussed above, significant advances have been made in the last decade in the fabrication of *p*-type, *n*-type, and *p*–*n* junction silicon films for PV applications by electrodeposition of SiO_2_ in CaCl_2_ molten salts. This trend will possibly continue in the future and eventually replace the production of conventional silicon wafers.

#### Electrochemical synthesis of various silicon-based materials

Besides the photoactive silicon films and nanostructured silicon discussed above, molten salt electrolysis has also been used as a template-free and simple green synthetic route to prepare some silicon components, such as silicide and SiC, which received considerable attention in recent years.

##### Silicides

Metal silicides have gained widespread applications due to their excellent high-temperature oxidation resistance and outstanding electrical/heat transfer properties [102,103]. As a potential new semiconductor material, Ca–Si alloy exhibits superconducting characteristics at high pressures with a critical temperature of 14 K [104–106]. Nevertheless, the high vapor pressure of Ca makes it extremely problematic to synthesize Ca–Si alloys by conventional chemical and vapor deposition methods. In contrast, preparing calcium silicide in CaCl_2_-based molten salts is simple. Ca–Si alloys can be produced through reaction (12) when the potential is more positive than the equilibrium potential *E*_Ca/Ca2+_ [24,65–68]. Sakanaka et al. [106] prepared Ca–Si films in molten CaCl_2_–KCl at 650 °C using a constant potential of −0.1 V. Subsequently, the multiphase Ca–Si films were converted to Si, CaSi, or CaSi_2_ phases by tuning the anodic potential. In addition, the authors elucidated various conversion reactions and the corresponding equilibrium potentials, which laid the foundation for the controlled preparation of calcium silicide.

$$y\mathrm{Si}+x{\mathrm{Ca}}^{2+}+2xe^{–}\to {\mathrm{Ca}}_{x}{\mathrm{Si}}_{y}$$ (12)

Recently, there has been an ever-growing interest in using intermetallic silicides to replace conventional graphite anodes in Li-ion batteries. These alloys used as anodes for Li-ion cells generally have lower capacities than pure silicon. Still, they show less volume change during lithiation/delithiation. Also, they maintain good structural stability and thus achieve better cycling. Various excellent results have been reported for the synthesis of silicon alloy anodes by molten salt electrolysis, such as Si–Fe [107], Si–Mn [108], Si–Cu [109], Si–Ti [110–112], and Si–Ge [113,114]. Zhou et al. [109] utilized SiO_2_ and Cu (molar ratio 1:1) as precursors to synthesize Cu–Si nanoalloys with a constant voltage of 2.4 V in molten CaCl_2_ at 700 °C. The main composition was Cu_9_Si and Si (denoted as Cu_9_Si/Si). During the electrolysis, SiO_2_ is continuously deoxygenated and reduced to Si. At the same time, Cu acts as a catalyst to promote the growth of Cu–Si nanowires along the Si axis, which exhibited good cycling stability and rate performance for lithium batteries. Ti-Si alloys were also prepared by direct electro-deoxidation of SiO_2_/TiO_2_ pellets (molar ratio 1:1) in molten CaCl_2_–NaCl at 700 °C, and the main product was nanoscale Si and Ti_5_Si_3_ (labeled as Ti_5_Si_3_/Si). Furthermore, Ti_5_Si_3_/Si demonstrated a good specific capacity of 638 mAh g^−1^ after 50 cycles at a charge/discharge rate of 200 mA g^−1^ [110]. However, the excess of Ti_5_Si_3_ in the product led to a decrease in lithium storage capacity. Hence, a superior anode is the TiSi_2_/Si nanoalloy, which can be prepared by adjusting the molar ratio of raw SiO_2_/TiO_2_ (20:1) [111]. Xiao et al. [113] prepared Ge–Si alloy nanotubes and hollow particles by direct electrolysis of GeO_2_ and SiO_2_ nanoparticles. The cavities formed benefited from the continuous solid diffusion controlled by the Kirkendall effect. The enhanced lithium storage performance of Ge–Si nanotubes fully illustrates the potential of alloy nanotubes.

##### SiC and Si/C composites

**Fig. 10.**
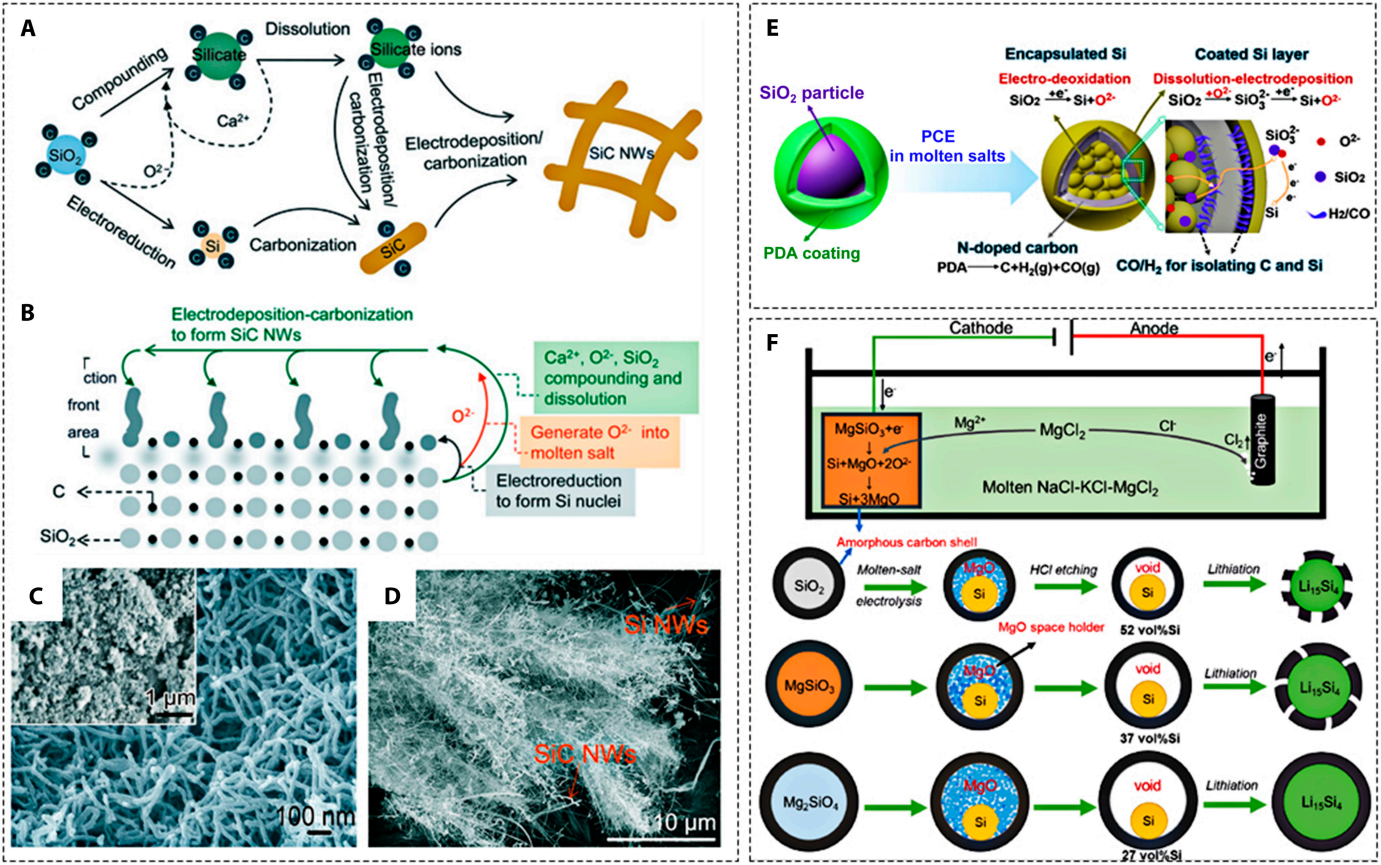
(A) Schematic diagram of the direct route from SiO_2_/C to SiC-NWs using a simple molten salt electrosynthesis strategy. (B) SLS mechanism of SiC-NWs formed from SiO_2_/C, including solid electroreduction, complexation–solubilization, and electrodeposition–carbonation processes. (C and D) SEM micrographs of SiC-NWs produced from SiO_2_/C powder by the SS mechanism and SiC-NWs/Si-NWs produced by the SLS mechanism. Reproduced from [103] with permission from Springer Nature. (E) Schematic diagram of the direct preparation of Si/C composites by pyrolysis–electrolysis of SiO_2_@PDA in molten salt. Reproduced from [120] with permission from the American Chemical Society. (F) Schematic diagram of the MgCl_2_-based molten salt electrolytic cell and the design of Si@void@C composites by the electrolysis-etching method. Reproduced from [121] with permission from the American Chemical Society.

SiC nanomaterials have been recognized as a new rising star [115]. Especially, SiC nanowires have attracted much attention due to their outstanding performance in many applications such as power [116] and harsh environment electronics [117], light detection devices [118], photocatalytic hydrogen production [119], and biochemical sensors [27]. Numerous works have reported that SiC can be extracted from molten chloride salts by direct electrolysis of SiO_2_/C or rice husk (RH). Yang et al. [72] obtained homogeneous SiC nanowires (SiC-NWs) by the electrolysis of SiO_2_/C (molar ratio 1:1) mixed pellets in molten CaCl_2_ at 900 °C with a constant voltage of 3.1 V. They proposed the corresponding reaction mechanism as well (Fig. 10A to C). Interestingly, SiC nanowires can also be produced by electrodeposition in molten CaCl_2_–SiO_2_/C. The reaction mechanism involves several processes: (a) composite formation, (b) dissolution, (c) electrodeposition, and (d) carbonization [Si + C → SiC, ∆*G*^0^= −63.94 kJ mol^−1^ (900 °C)] (Fig. 10B). However, since the Si nuclei cannot be surrounded by the carbon powder dispersed in molten CaCl_2_, the products are usually a mixture of Si-NWs and SiC-NWs (Fig. 10D). Obviously, these results provide an essential guideline for the controllable preparation of SiC nanostructures or Si/C composites by molten salt electrochemical processes.

Silicon and carbon composites are known to be ideal anode materials for Li-ion batteries. Introducing highly conductive carbon not only reduces the polarization of silicon particles but also increases the material’s electrical conductivity, thus improving the cycling and rate performance of the battery. Such advantages of Si/C composites motivated the direct preparation of Si/C composites by molten salt electrolysis. However, Si produced by reducing SiO_2_ at high temperatures would react spontaneously with C to form SiC. Thus, the preparation of Si/C composites based on molten salt electrochemistry has been deemed to be a challenging task.

The strategy of preparing Si/C composites by simultaneous pyrolysis and electrolysis of polydopamine (PDA) encapsulated SiO_2_ nanoparticles in molten salts was proposed and validated [120]. During the reaction, SiO_2_ is reduced to Si via electro-deoxidation. Simultaneously, PDA is pyrolyzed to obtain N-doped carbon. Notably, the gases (CO, H_2_) generated during the pyrolysis will act as a physical barrier at the interface between Si and C, effectively inhibiting the formation of SiC. In addition, the ionized oxygen tends to react with the neighboring SiO_2_ to form SiO_3_^2−^, which then produces new Si on the outer surface of carbon via an SLS mechanism, leading to the formation of Si@C@Si microstructures with enhanced lithium storage capacity (Fig. 10E). Alternatively, the use of MgCl_2_-based molten salts is also considered to be efficient in preventing the formation of SiC. Recently, it has been shown that core-shell Si/C composites can be prepared directly by electrochemical reduction of C@SiO_2_ in molten NaCl–KCl–MgCl_2_ at 650 °C [121,122]. The O^2−^ produced during electrolysis reacts with Mg^2+^ to form MgO, which has low solubility in molten NaCl–KCl–MgCl_2_. MgO can prevent the spontaneous reaction of Si with C at high temperatures. Besides, removing MgO by water following the reaction would form voids within the carbon shell. The space formed can act as a buffer for the volume expansion of the Si/C anode material during the lithiation/delithiation process. If more cavities are needed within the carbon shell, carbon-coated magnesium silicate (C@MgSiO_3_, C@Mg_2_SiO_4_) can be employed to generate more MgO in situ during electrolysis (Fig. 10F).

**Fig. 11.**
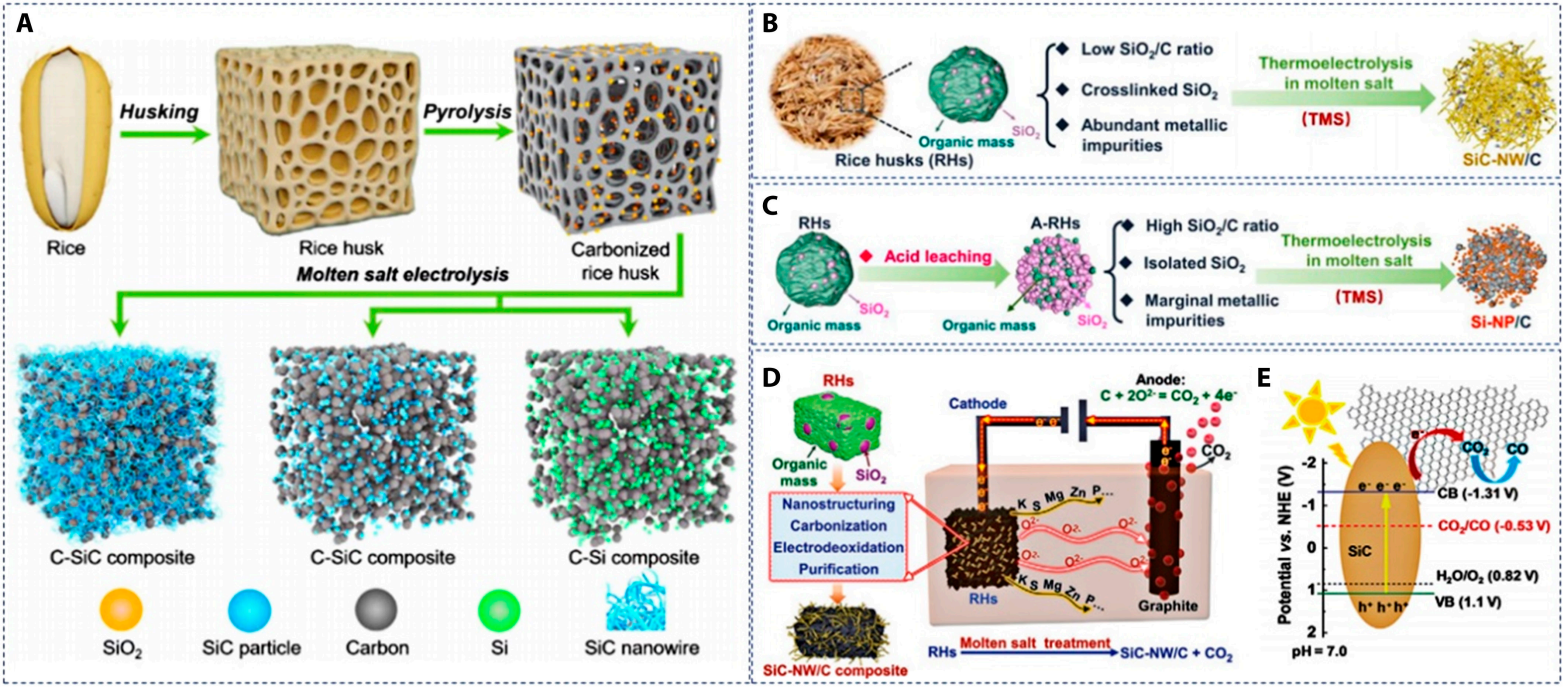
(A) The preparation of C/SiC and C/Si composites from RHs by molten salt electrolysis under constant potential values. Reproduced from [128] with permission from Wiley-VCH. (B) Schematic diagram and associated SEM and TEM micrographs of RHs with highly cross-linked structures with low SiO_2_/C molar ratio and high content of metal impurities converted to SiC-NW/C. (C) Schematic diagram and associated SEM and TEM micrographs of the A-RHs containing isolated SiO_2_, high SiO_2_/C molar ratio, and marginal metal impurities converted to Si-NP/C. Reproduced from [129] with the permission of Elsevier. (D) Reaction process of molten salt-assisted electrochemical conversion of RHs to SiC-NW/C. (E) Illustration of CO_2_ photoreduction process over SiC-NW/C. Reproduced from [130] with permission from Wiley-VCH.

RHs are typical agricultural waste with an annual production of up to 100 million tons. It contains 15 to 20 wt.% SiO_2_, 75 to 85 wt.% organic matter, and other trace inorganic elements (such as Mg, Zn, K, S, and P) [123–125]. Therefore, converting RHs into SiC or Si/C composites is an attractive option, i.e., it achieves the utilization of both components and avoids the waste of resources [126,127]. Zhao et al. [128] investigated the electrochemical conversion of RHs to SiC and SiC composites in CaCl_2_ or NaCl–KCl–MgCl_2_ molten salts. Before electrolysis, the RHs were first converted by pyrolysis from amorphous hydrated silica and organic matter to SiO_2_ and hard carbon, respectively. The SiO_2_/C composites were then electrochemically converted at a constant voltage of 2.4 to 2.8 V to obtain different products (Fig. 11A). At 2.4 V, randomly distributed SiC nanowires were obtained on amorphous carbon particles. At a higher voltage of 2.6 V, the product was a mixture of honeycomb SiC nanowires and carbon particles. When the electrolysis voltage was increased to 2.8 V, carbon flakes and interconnected nanowires were observed. Moreover, the authors found that lower potential is favorable to hindering SiC formation. Thus, Si/C composites can be obtained in NaCl–KCl–MgCl_2_ molten salts with electrolytic voltages below 2.5 V, which has excellent rate performance for Li-ion battery, maintaining a high capacity of 926 mAh g^−1^ even at a current density of 500 mA g^−1^. However, the fact that pyrolysis and electrolysis are carried out in 2 steps complicates the whole reaction route and reduces the treatment efficiency.

Pang et al. [129] argue that CaCl_2_-based molten salts are still a better choice for the electrochemical reduction of SiO_2_ than MgCl_2_-based ones. The reason behind this is the sustained and efficient conversion and the reduced anodic evolution of corrosive Cl_2_. Concerning the uncontrollable formation of SiC in CaCl_2_-based molten salts, the authors propose that this can be countered by precise modulation of the microstructure and composition of RHs. The feasibility of controlling the conversion of SiO_2_ and organic matter in RHs to form SiC/C or Si/C by superheated electrolysis in CaCl_2_–NaCl molten salts has also been verified. RHs with a highly cross-linked structure with a low SiO_2_/C molar ratio and high content of metal impurities were converted to SiC-NW/C. The product exhibited potential as microwave-absorbing material (Fig. 11B). Acid-impregnated RHs (A-RHs) containing isolated SiO_2_, with high SiO_2_/C molar ratio and marginal metal impurities, were converted to Si–NP/C (Fig. 11C). This was attributed to the bubble phenomenon caused by CO and H_2_ released in the molten salt during the thermal electrolysis of organic matter that effectively hindered SiC formation. Nevertheless, the process described earlier yields greenhouse gases (CO_2_ emissions). The same team recently reported that the SiC-NW/C obtained from RHs could be used as an efficient CO_2_ reduction photocatalyst [130]. As shown in Fig. 11D, RHs are electrochemically converted to SiC-NW/C and CO_2_ in molten CaCl_2_–NaCl. Due to the strong coupling between the components of SiC-NW/C, the photoelectrons generated by SiC-NW through excitation by light absorption will rapidly migrate to the porous carbon. The conversion of the adsorbed CO_2_ to CO occurs (Fig. 11E), providing sustainable power for the molten salt electrolyzer. Hence, the conversion of RHs to SiC-NW/C is theoretically a closed-loop carbon cycle bridge in abundant light. Undoubtedly, these studies provide new insights into the direct access to SiC or Si/C mixtures from RHs by molten salt electrolysis while opening a new path for the sustainable production of Si-related energy storage materials.

### Silicon electrodeposition in room-temperature ionic liquids

#### Ionic liquids—An alternative to high-temperature molten salts?

Ionic liquids are molten salts with melting points below 100 °C, which consist of only cations and anions [131,132]. Compared to conventional simple metal halides (e.g.*,* AlCl_3_), the cations and anions of ionic liquids are relatively complex. The charges are often delocalized or shielded by side groups [133]. Besides, ionic liquids exhibit good electrical conductivity, a wide electrochemical window (>5 V), and high thermal stability, so they can be used to electrodeposit silicon at room or milder temperatures. In this case, ionic liquids are called “room-temperature molten salts” and are considered an alternative to high-temperature molten salts [20].

Reportedly, Katayama et al*.* [134] found that silicon can be electrodeposited at low temperatures (90 °C) in ionic liquids (1-ethyl-3-methylimidazolium hexafluorosilicate). However, the product completely converted to SiO_2_ when exposed to air. Thus, it remained an open question whether the obtained silicon had semiconducting properties. In 2004, El Abedin and colleagues [135,136] reported for the first time that efficient nanoscaled electrodeposition of elemental silicon was achieved at room temperature using SiCl_4_ as the silicon source in 1-butyl-1-methylpyrrolidinium bis(trifluoromethylsulfonyl)imide ([BMP][Tf_2_N]). However, the purity of the deposits was unsatisfactory. The authors found that electrodeposition can severely disturb interfacial processes even at a ppm concentration atmosphere. Besides, silicon nanoparticles easily interact with metal substrates during deposition to form intermetallic silicides. Recently, Tsuyuki et al. [137] have also tried to electrodeposit silicon thin films for solar cells in ionic liquids and systematically investigated the doping of the film structure. *N*-trimethyl-*N*-hexylammonium bis(trifluoromethanesulfonyl)imide (TMHA-TFSI) and SiCl_4_ were used as the ionic liquid and silicon source, respectively, to promote the growth of continuous and dense silicon films by adjusting the light time. The addition of AlCl_3_ resulted in the formation of *p*-type silicon films. However, these films were primarily oxidized, which may be attributed to the films’ exposure to air or during annealing in an attempt to remove impurities generated by ionic liquids.

**Fig. 12.**
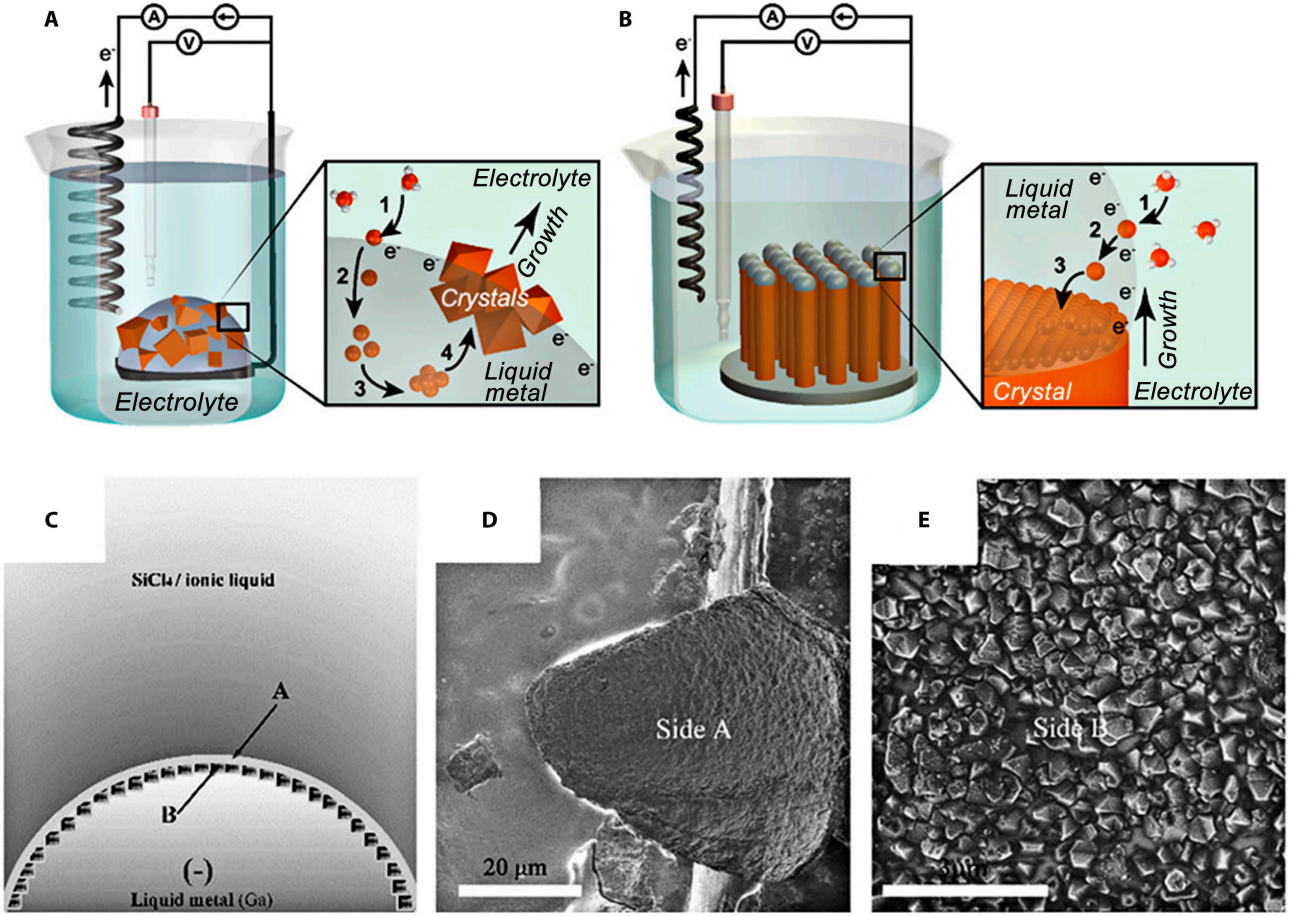
Schematic diagram of the experimental setup and ec-LLS steps for growing semiconductors from (A) bulk and (B) nano/micro-scale liquid metal-droplet electrodes. Reproduced from [139] with permission from the American Chemical Society. (C) Illustration of the fabrication of crystalline silicon films on the surface of liquid Ga in ionic liquids with SiCl_4_ as a precursor. (D and E) SEM of polysilicon particles on side A (D) and amorphous silicon on side B (E) as labeled in (C). Reproduced from [140] with permission from the Royal Society of Chemistry.

Generally, a high temperature (>700 °C) is required for the electrodeposition process to produce crystalline silicon. While prepared at low temperatures, it is always amorphous and requires additional thermal annealing and purification. Gu et al*.* [138] proposed a new strategy for electrochemical synthesis using a liquid metal electrode (e.g., Ga). The latter can act as a source of electrons for dissolved species and as a recrystallization solvent. This strategy is called “electrochemical liquid–liquid–solid” (ec-LLS) crystal growth (Fig. 12A and B), which allows the direct production of crystalline silicon at near ambient temperatures [139]. Recently, Zhang et al*.* [140] reported an efficient method to fabricate crystalline silicon films using SiCl_4_ on a liquid gallium surface at low temperatures in the presence of tri-1-butylmethylammonium bis((trifluoromethyl)sulfonyl)amide ([N4441][TFSI]) as the electrolyte. The silicon films had 2 sides, one containing polycrystalline silicon particles and the other smooth amorphous silicon (Fig. 12C to E). The authors suggested that the possible growth mechanisms for crystalline Si include the formation of liquid Si in liquid Ga followed by nucleation and growth of crystalline Si on reduced amorphous Si films. Zhao et al*.* [141] obtained excellent-quality crystalline silicon films in a similar system by adjusting the reaction temperature, deposition potential/duration, and different substrates. However, as an attractive strategy for the fabrication of crystalline Si in low-temperature ionic liquids, ec-LLS strategies still have many problems to be addressed.

As discussed above, considerable progress has been made in the electrodeposition of silicon in ionic liquids. However, the incompatibility of low-temperature and pure crystalline products severely limits the attractiveness of the process. In the future, the silicon oxidation contamination problem deserves further attention. Moreover, further investigation into the controllable deposition and application of liquid electrodes is necessary to facilitate the development and popularization of this strategy for producing crystalline silicon at room temperature.

#### New opportunities for designing Si structures: Controlled tuning of templates and parameters

**Fig. 13.**
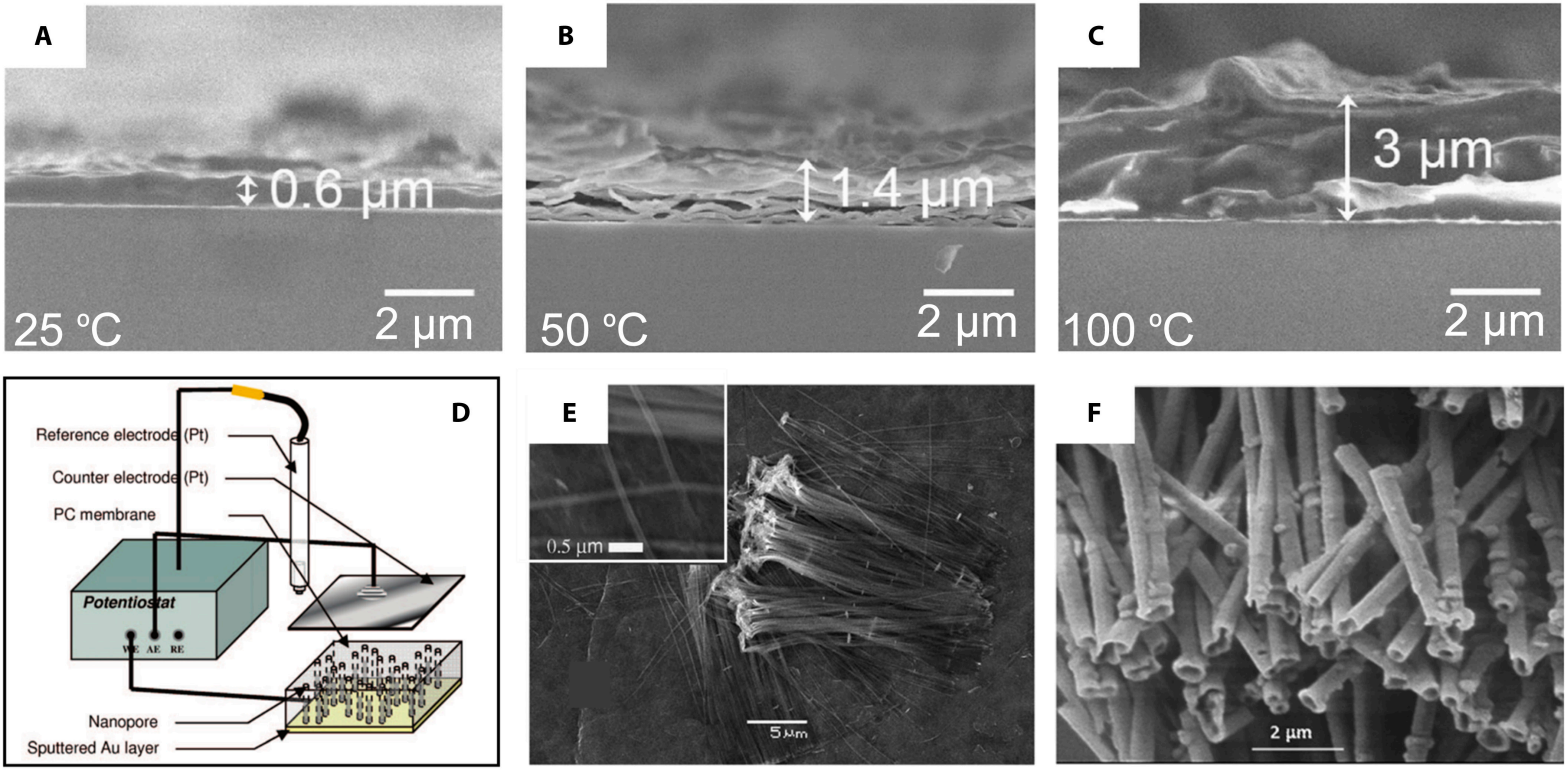
Cross-sectional SEM micrographs of silicon electrodeposited from electrolytes containing Py1,4[TFSI] and SiCl_4_ at (A) 25 °C, (B) 50 °C, and (C) 100 °C. (D) Schematic diagram of the electrodeposition apparatus used to prepare 1-dimensional nano-Si. (E) SEM micrographs of Si-NWs were prepared using a membrane with a pore size of 110 nm and a thickness of 20 μm. Reproduced from [144] with permission from the American Chemical Society. (F) SEM micrograph of 400-nm Si-NTs grown in 400-nm polycarbonate (PC) membranes. Reproduced from [143] with permission from the Royal Society of Chemistry.

Electrodeposition from room-temperature ionic liquids offers a potential alternative to traditional physical vapor or chemical vapor deposition as a cost-effective technique in preparing class IV thin films or nanostructures such as silicon. Thomas et al*.* [142] described the effects of potential, the concentration of electroactive substances, temperature, and organic additives on silicon deposits in 1-butyl-1-methylpyrrolidinium bis(trifluoromethanesulfonyl)imide ionic liquids. It was found that the concentration of electroactive substances or additives in the solvent has an almost negligible effect on the synthesis of silicon. However, the temperature and the applied potential were crucial. The cross-sectional morphology of the silicon films at different temperatures (25, 50, and 100 °C) is interesting (Fig. 13A to C). Compared to silicon films deposited at room temperature, the one obtained at 100 °C is rougher and exhibits lower adhesion to Au substrates, indicating that temperature plays a crucial role in silicon growth. Thus, the deposition of silicon films at 50 °C can be balanced by adjusting the deposition rates, adhesion, and growth roughness. Furthermore, the authors note that prolonged electrodeposition of silicon in ionic liquids will be limited by the maximum charge that can be passed. However, the charge limitation can be improved by increasing the temperature, potential, or concentration of electroactive substances. This study offers new insights into silicon electrodeposition in room-temperature ionic liquids. This is crucial for controlling and improving the synthesis of silicon for specific applications.

An exciting aspect of silicon electrodeposition in room-temperature ionic liquids is the ability to grow differently shaped nanomaterials in a template-assisted way. Mallet and colleagues [143,144] obtained pure amorphous Si-NWs and Si-NTs by electrodeposition at room temperature using SiCl_4_ as the precursor in 1-butyl-1-methylpyrrolidinium (trifluoromethylsulfonyl) imide (P_1,4_) (Fig. 13D and E). The diameter or wall thickness of the deposit can be precisely controlled by simply changing the electrodeposition parameters or the size of the template (polycarbonate film). Annealing can crystallize silicon deposits without changing their shape and structure. Such a strategy may replace the more restrictive high-vacuum approach, and the synthesized homogeneous silicon nanomaterials have promising applications in microelectronics and optoelectronics [145,146].

Some recent publications have demonstrated that synthesizing ionic liquids can yield Si-based anode materials by surface plating or co-deposition at room temperature [147–149]. Kowalski et al. [147] deposited a continuous, compact silicon shell on the surface of TiO_2_ nanotubes via electrodeposition from SiCl_4_ in room-temperature ionic liquid. The thickness of the silicon shell was adjustable by the charge density. Silicon layer thicknesses of 20, 60, and 100 nm can be obtained at charge densities of 0.3, 1.0, and 1.8 C cm^−2^, respectively. Li et al. [150] fabricated porous sheet-like Si–Cu composites. Its structure contained alternating Si–Cu layers on a Cu substrate. The applied potential was 1.9 to 2.1 V during electrodeposition in ionic liquids containing 0.002 mol l^−1^ Cu(TfO)_2_ and 1 mol l^−1^ SiCl_4_-[BMP]Tf_2_N. The porous structure was caused by the SiCl_4_ bubbles attached to the Si–Cu films. The authors pointed out that introducing copper helps to obtain a specific silicon morphology and improves its conductivity. The as-obtained Si–Cu porous sheets used as an anode material have a capacity of 1,042.8 mAh g^−1^ after 600 cycles at a current density of 21 A g^−1^.

## Challenges and Potential Solutions

**Fig. 14.**
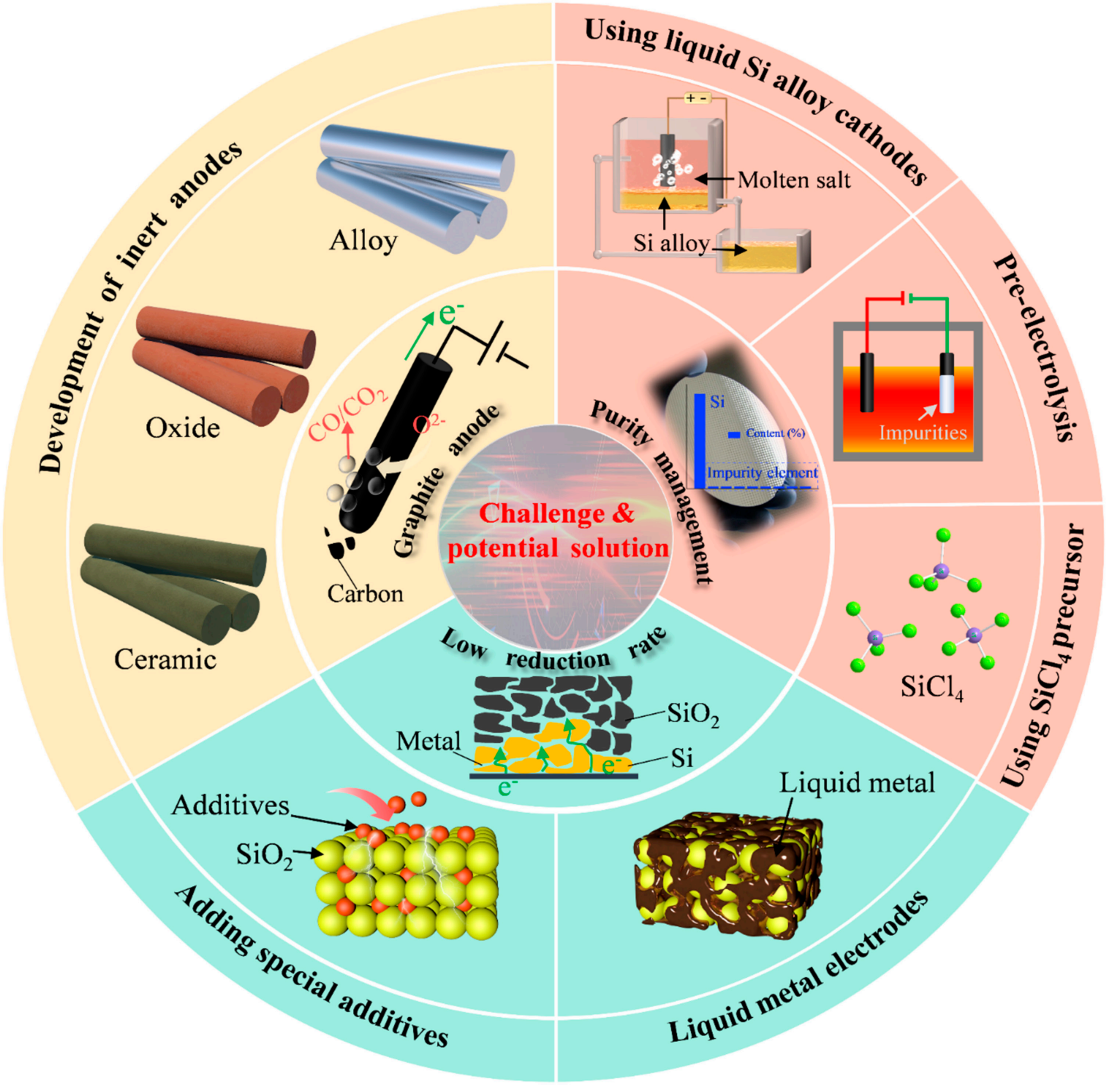
Challenges and strategies for the electrochemical production of silicon in molten salts.

Notwithstanding the recent promising progress in silicon electrochemistry research, several scientific and technical obstacles remain to achieve practical applications, including developing inert anodes, improving electroreduction rates, and production of high-purity silicon. Hence, the challenges of silicon electrochemistry are summarized in Fig. 14, and potential solutions are presented to advance silicon electrolysis in real-world applications.

### Development of inert anodes

Graphite has been widely employed as an anode material for high-temperature molten salt electrochemical extraction of silicon due to its excellent electrical conductivity and thermal stability [151]. However, there are several effects on the graphite anode during the electrochemical production of silicon: (a) O^2−^ released from the silica or silicate during reduction will inevitably react with the graphite anode, generating undesirable CO or CO_2_ [152]; (b) CO and CO_2_ will partially dissolve in the molten salt, generating CO_3_^2−^ and reducing at the cathode, resulting in a significant decline in the purity and performance of the product silicon; (c) the carbon powder peeled off from the graphite anode will float on the surface of the molten salt during long-term electrolysis, thus causing a short circuit between the cathode and anode. A daunting challenge is developing stable, inert anodes not consumed in high-temperature molten salts.

Various prospective anode materials have been used to assemble molten salt electrolysis, including alloys, oxides, and ceramics [153–160]. The Cr–Fe alloy anode seems promising because it shows excellent stability in electrolytic processes involving molten iron at temperatures up to 1,565 °C [153]. This is a crucial reference for silicon electrolysis at even lower temperatures. Another potential anode material is CaTiO_3_/CaRuO_3_-based composite, which shows extremely low corrosion rates and oxygen precipitation in CaCl_2_-based molten salts [158,159]. However, the usage of CaRuO_3_-based anodes may be restricted by the limited reserves of Ru. Recently, a Magnéli-phase titanium oxide (Ti_4_O_7_) inert anode with conductivity close to that of graphite was obtained, which produced only oxygen during silicon electrolysis [160]. More importantly, this anode can be regenerated by current reversal or chemical reduction of H_2_ in the molten salt, thereby enabling recycling. However, such excellent performance was merely tested under laboratory conditions, and many of the characteristic values obtained are unrepresentative of actual production conditions.

### Improvement of electroreduction rate

The reduction rate is an important indicator to determine the feasibility of a silicon electrochemical extraction technology. In the case of electrodeposition, continuous electrolysis will result in a significant decrease in the silica reduction rate because the deposited silicon precipitates as a solid on the substrate. To address this challenge, an experimental approach was suggested in which the deposition of liquid silicon should be carried out in the BaO–SiO_2_–BaF_2_ system [39]. However, the relatively high melting point of silicon (1,414 °C) is a drawback that hinders the scaling up of the process. However, it is not impossible to overcome this obstacle. For example, its melting point is much lower than that of iron. Regarding the silicon electro-deoxidation technology, the reduction rate of SiO_2_ is extremely low due to the deoxidation reaction mainly occurring near the 3PIs. Even porous particles pressed from SiO_2_ (2 to 7 μm) require several hours to achieve complete reduction [65]. Future research may focus on 2 directions: (a) Finding a suitable additive that not only improves the electrical conductivity of SiO_2_ precursor but also allows complete separation from the product at the end of the reaction, and (b) exploring appropriate liquid metal electrodes instead of common ones would significantly increase the contact area between the electrode and the silica precursor and ensure stable electrochemical reduction at high current densities.

### Production of high-purity Si

The key challenge in silicon electrochemistry is purity, as the silicon purity for PV devices is at least 6 N (≥99.9999), while that used in electronic devices is higher (EG-Si or 11 N). In most cases, silicon precursors, reaction vessels, electrode-connected metals, electrolytes, anodes, and cathode materials can introduce impurities during the electrochemical reduction process. Without a one-step purification, the reduced silicon will hardly meet the above requirements.

Pre-electrolysis is a typical purification method because it effectively reduces impurities in the electrolyte. When silicon precursors are present (e.g., fluorosilicate/fluoride systems), impurities with reduction potentials lower than silicon can be removed by setting a pre-electrolysis potential. However, impurities with a reduction potential higher than the pre-electrolysis potential remain in the electrolyte. In this regard, Xu and Haarberg [26] suggested that precise regulation of the concentration of electroactive substances and the applied potential can improve the purity of the product. In the case of SiO_2_ or CaSiO_3_ as precursors, there is an opportunity to periodically pre-electrolyze the electrolyte so that all impurities are decreased to tolerable thresholds (e.g., Mg < 0.05 ppm, Na < 0.05 ppm, and W < 0.05 ppm). This was confirmed by Zou et al. [73] as they obtained ~6 N purity silicon films.

Nohira’s research team suggested that using gaseous SiCl_4_ as a silicon precursor for the electrochemical extraction of high-purity Si might be very promising [161] since the well-known Siemens purification technology also employed a gaseous silicon source (SiHCl_3_). Although SiCl_4_ is almost insoluble in chloride electrolytes, its solubility in KCl–KF has exceeded 80%. It has the advantage that the anode reaction is the evolution of Cl_2_ rather than the emission of CO or CO_2_. Moreover, the generated Cl_2_ can be collected and used for the chlorination of SiO_2_ and then distilled to prepare high-purity SiCl_4_. In addition, using liquid Si to control the impurity content is also a promising means of purification. During the separation of the solid Si from the liquid alloy, the impurities will remain in the liquid phase. Yasuda et al. [162] proposed that using liquid Si–Zn cathodes to produce SoG-Si in molten CaCl_2_, the molten salt effectively inhibited the evaporation of Zn, thus ensuring the long-time operation of the Zn cathodes. The reduction of SiO_2_ on Zn proceeds at *E* < 1.45 V (versus Ca^2+^/Ca), which is much more positive than the reduction of SiO_2_ on Si. Pure Si particles can be collected during the cooling of the Si–Zn system.

## Summary and Outlook

### Advantages and drawbacks/limitations of various strategies

**Fig. 15.**
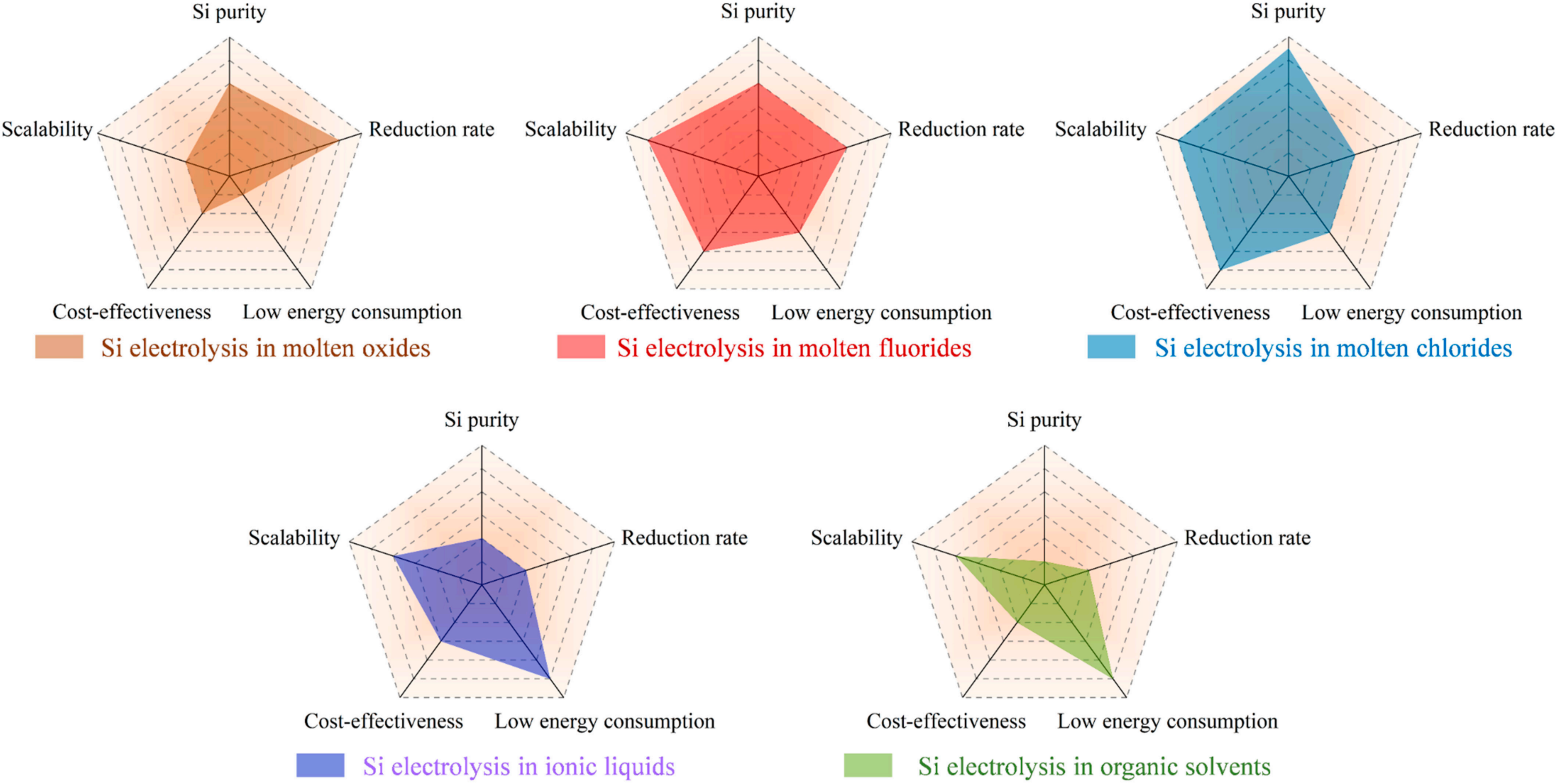
A summary of the advantages and drawbacks/limitations of different electrochemical extraction strategies for Si.

Silicon electrochemistry is a constantly evolving field that provides eco-friendly and energy-efficient silicon extraction and processing strategies. This paper reviews the history and recent advances in the electrochemical production of silicon. As discussed in the review, several promising strategies have been attempted. Each has its advantages and drawbacks, so the characteristics (Si purity, scalability, cost-efficiency, reduction rate, and low energy consumption) of these strategies will be summarized from the point of view of the electrolytes (Fig. 15).

The electrolysis of Si in molten oxide is also referred to as the electrowinning of liquid Si. Since Si is electrodeposited in liquid form, it is reduced at a speedy rate. As shown in Table, the purity of the silicon ingots obtained from the barium silicate solution is about 99.98 wt.%. This purity is close to the quality required to produce solar cells with 10% efficiency by a single directional solidification stage. However, the applied ultra-high temperatures (~1,400 °C) also result in high energy consumption and limitations in the choice of equipment materials, making it difficult to scale up.

**Table.** Experimental conditions and representative product characteristics of different silicon electrochemical extraction strategies.

Electrolyte	Precursor	Temperature	Concentration	Substrate/contacting electrode	Morphology	Purity	Reference
BaO–SiO_2_–BaF_2_	SiO_2_	~1,400 °C	63.2 mol%	Graphite	Ingot	~4 N	[39]
LiF–KF/LiF–NaF–KF	K_2_SiF_6_	600–850 °C	1–10 mol%	Si (single crystal)	Columnar grain	/	[41,42]
LiF–KF/LiF–NaF–KF	K_2_SiF_6_/Na_2_SiF_6_	745–850 °C	0.05–16 mol%	Graphite/Ag	Dendrite/film	/	[43]
LiF–NaF–KF	Na_2_SiF_6_	820–900 °C	0.24 mol%	Graphite/Ni/Ag	Microwire/sponge/film	/	[48]
KF–KCl	K_2_SiF_6_/SiCl_4_	650–800 °C	0.5–5 mol%	Graphite/Ag	Nodular/film	~4 N	[50–52]
KF–KCl	K_2_SiF_6_	650 °C	1 mol%	Graphite	Film	/	[53]
KF–KCl–KI	K_2_SiF_6_	725 °C	0.075–0.5 mol%	Graphite/W	Film	/	[54]
TBAB–PC	SiHCl_3_	22 °C	0.2–0.5 mol%	Au	Film	/	[56]
TEAC/TPAC/TBAC/TPnAC/THAC	SiHCl_3_	35–145 °C	0.02–1 mol%	Pt/Ti/Ti-6Al-4V alloy/n-Si/ITO	Film	/	[58]
PC–TBAC	SiCl_4_	130 °C	0.5 mol%	Ni	Film	/	[61]
TBAC/CH_3_CN/THF	SiCl_4_/SiHCl_3_	350–800 °C	0.3 mol%	Ni/Ag/GC	Film	/	[62]
CaCl_2_	Quartz	850 °C	/	Mo	Hexagonal column	/	[24]
CaCl_2_	SiO_2_	900 °C	/	W	Nanoparticle	/	[65–67]
CaCl_2_–CaO	SiO_2_/CaSiO_3_	850 °C	3.9 mol%/1–2.2 wt.%	Graphite	Film	~6 N	[72,73]
CaCl_2_	Quartz	850 °C	/	Mo	Nanowire array	/	[82]
CaCl_2_–MgCl_2_–NaCl	CaSiO_3_	650 °C	1 wt.%	Graphite	Nanowire	/	[84]
CaCl_2_–NaCl	SiO_2_/CaSiO_3_	800 °C	/	Ni/graphite	Nanotube	/	[87–89]
CaCl_2_	SiO_2_	850 °C	/	Ag	Film	~3N	[99]
[BMP][Tf_2_N]	SiCl_4_	25 °C	0.1 mol%	Pt/Au	Nanoparticle	/	[135,136]
[TMHA][TFSI]	SiCl_4_	25 °C	0.5 mol%	Pt	Film	/	[137]
[N_4441_][TFSI]	SiCl_4_	100 °C	0.25–0.5 mol%	Ga	Film	/	[140]
[BMP][Tf_2_N]	SiCl_4_	25 °C	0.1 mol%	Au (PC template)	Nanotube	/	[143]
[BMP][Tf_2_N]	SiCl_4_	25 °C	/	Au (PC template)	Nanowire	/	[144]

PC, propylene carbonate; TBAB, tetrabutylammonium bromide; TEAC, tetraethylammonium chloride; TPAC, tetrapropylammonium chloride; TBAC, tetrabutylammonium chloride; TPnAC, tetrapentylammonium chloride; THAC, tetrahexylammonium; THF, tetrahydrofuran; [BMP][Tf_2_N], 1-butyl-1-methylpyrrolidinium bis(trifluoromethylsulfonly)imide; TMHA-TFSI, *N*-trimethyl-*N*-hexylammonium bis(trifluoromethanesulfonyl)imide; [N_4441_][TFSI], tri-1-butylmethylammonium bis((trifluoromethyl)sulfonyl)amide.

Molten fluoride is recognized as the most promising electrolyte for Si electrodeposition because of its inherent etching properties. The most extensively investigated systems are fluorosilicates dissolved in molten fluoride salts (e.g.*,* LiF, NaF, and KF). The electrolysis temperature is generally between 450 and 800 °C, with relatively low energy consumption. Fluorosilicates have been thoroughly studied as precursors, but their continued availability as an inexpensive source depends on the fertilizer industry. However, fluorosilicates can also be synthesized in situ by SiO_2_ with alkaline earth fluoride baths. Although it offers significant advantages in terms of cost-effectiveness and energy efficiency, it involves problems mainly linked to highly corrosive HF, including control of impurity levels, severe Si pitting, and surface staining and clouding of Si. The purity of Si obtained in fluoride–chloride electrolytes (e.g.*,* KCl–KF) is only around 4 N.

Chloride molten salts are the most widely utilized electrolytes, especially CaCl_2_, which has been intensively studied for the electrochemical reduction of silica as an environmentally friendly, water-soluble, and inexpensive salt. The temperature of electrolytic Si in molten chloride is generally 500 to 900 °C, so the energy consumption is relatively low. The solid-to-solid reaction drives the electroreduction process of SiO_2_, and the reduction reaction occurs mainly near the 3PIs, so the reduction rate is very slow. Its potential ways of improvement have been mentioned in the “Improvement of electroreduction rate” section. Despite its very low solubility in molten chloride salts, SiO_2_ can also be reduced by an SLS reaction in CaCl_2_-based molten salts containing CaO. This method is based on a dissolution–electrodeposition mechanism. SiO_2_ is dissolved initially in CaCl_2_ with a high concentration of O^2−^, and then Si is electrodeposited from the resulting silicate ions. This strategy has now been shown to be helpful in depositing Si films with purity up to nearly 6 N.

Ionic liquids and organic solvents used as electrolytes opened the door to silicon electrolysis at room temperature. They are very economical and convenient compared to high-temperature electrolysis techniques. The main advantages of ionic liquids are their good electrical conductivity, high thermal stability, and wide electrochemical window (>5 V), which allows them to be used for the electroreduction of Si at room temperature. Besides, ionic liquids are highly safe and eco-friendly due to their low vapor pressure, non-flammability, non-toxicity, and biodegradability. The advantages of organic solvents also include a wide electrochemical window, which makes them sufficient for the electroreduction of Si. However, Si conducts poorly at low temperatures, and the Si deposited on the substrate surface will act as an insulating layer hindering the continuous reduction of Si. In addition, Si deposited at room temperature is susceptible to oxidation in air and incorporation into other substances in the electrolyte in organic solvents; thus, the purity is low.

### Future directions

#### Electrodeposited Si film for PV cells

**Fig. 16.**
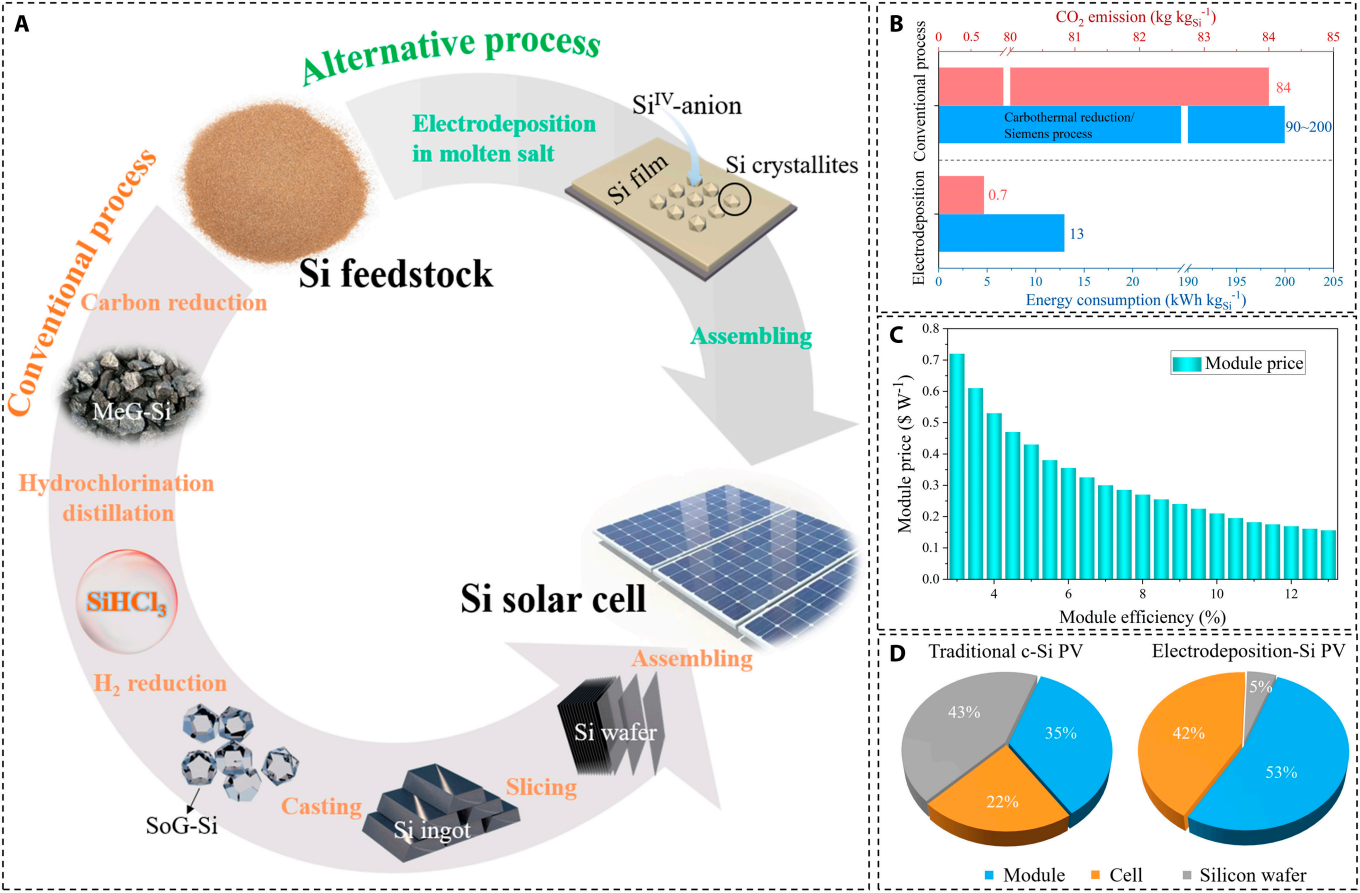
(A) Comparison of the molten salt electrodeposition strategy and the conventional process for the fabrication of SoG-Si. (B) CO_2_ emissions and energy consumption for the production of silicon for solar cell by different methods (these data were obtained from [21,160]). (C) Cost analysis based on SiO_2_–CaO–CaCl_2_ electrodeposition technology. Manufacturing cost per watt of solar cell module versus module efficiency. (D) Cost breakdown comparison of electrodeposited silicon PV (10% power conversion efficiency) and conventional crystalline silicon (c-Si) PV (21% power conversion efficiency). (C) and (D) are reproduced from [73] with permission from Springer Nature.

In principle, electrodeposition of silicon from molten salts is highly cost-effective. The reason behind it is the production of silicon wafers for solar cells directly from raw materials in a single-stage process, which is unmatched by any competing process (Fig. 16A). In terms of feedstock and product, the electrochemical production of silicon is analogous to industrial carbothermal deoxidation reduction. However, the carbothermal reduction steps require significant energy consumption and emit huge amounts of CO_2_ (Fig. 16B). For reference, each kilogram of polysilicon consumes approximately 20 kWh of electricity and emits at least 84 kg of CO_2_ [21]. An additional 90 to 200 kWh kg_Si_^−1^ is required when further purification is carried out using the Siemens process. In contrast, the electrical energy for the electrodeposition of silicon would be reduced to approximately 13 kWh kg_Si_^−1^. The CO_2_ emissions from silicon electrodeposition are mainly associated with the most commonly used graphite anodes (already discussed in the “Development of inert anodes” section), which are reported to consume approximately 0.19 kg of carbon and emit 0.7 kg of CO_2_ for every 1 kg of polysilicon produced [160]. Despite this challenge, the CO_2_ emission is significantly lower than the industrial methods.

The fluorosilicate/fluoride system is ideal for the direct electrodeposition of silicon films because of its cost and energy efficiency. Nevertheless, this process is still a long way from actual industrial production due to the high aggressiveness of fluorides. Those compounds make it difficult to control the level of impurities in the deposit. Electrolysis of the SiO_2_–CaO–CaCl_2_ system has been considered a promising strategy for depositing high-purity silicon and *p*-type, *n*-type, and *p*–*n* junction silicon films. Efforts toward achieving better photoactive silicon films have intensified over the past decade. In particular, Bard’s team has assembled PV cells with near-solar grade (SoG-Si) purity with a power conversion efficiency of 3.1%, confirming the potential of this strategy for real-world applications. Figure 16C summarizes the manufacturing cost per watt of solar cell module versus module efficiency based on electrodeposition technology [163–165]. The production cost of electrodeposited silicon wafers is significantly lower than that of conventional methods (Fig. 16D), and a module cost of nearly 0.2 $Wp^−1^ can be achieved by improving the energy conversion efficiency of the cell to 10%. These data show the potential of electrochemical methods for the direct preparation of low-cost solar cells; however, its processes for practical applications still need to be significantly developed. It is worth mentioning here that despite recent good progress in the electrochemical production of silicon with a purity close to solar grade, the method is still a long way from producing higher-purity silicon (such as EG-Si) and will hardly be able to compete with silicon produced by the semiconductor industry in a short term. Improving purity is therefore a key challenge for future work. Amorphous silicon, a prospective material for large-scale PV devices, can, in principle, be fabricated by low-temperature electrodeposition from ionic liquids and organic solvents. Still, research to date looks less promising than alternative molten salt processes that yield crystalline silicon. A primary reason is the low conductivity of silicon at low temperatures, which limits electrodeposition and prevents it from forming a continuous dense film.

#### Electrochemical synthesis of various Si-based materials

**Fig. 17.**
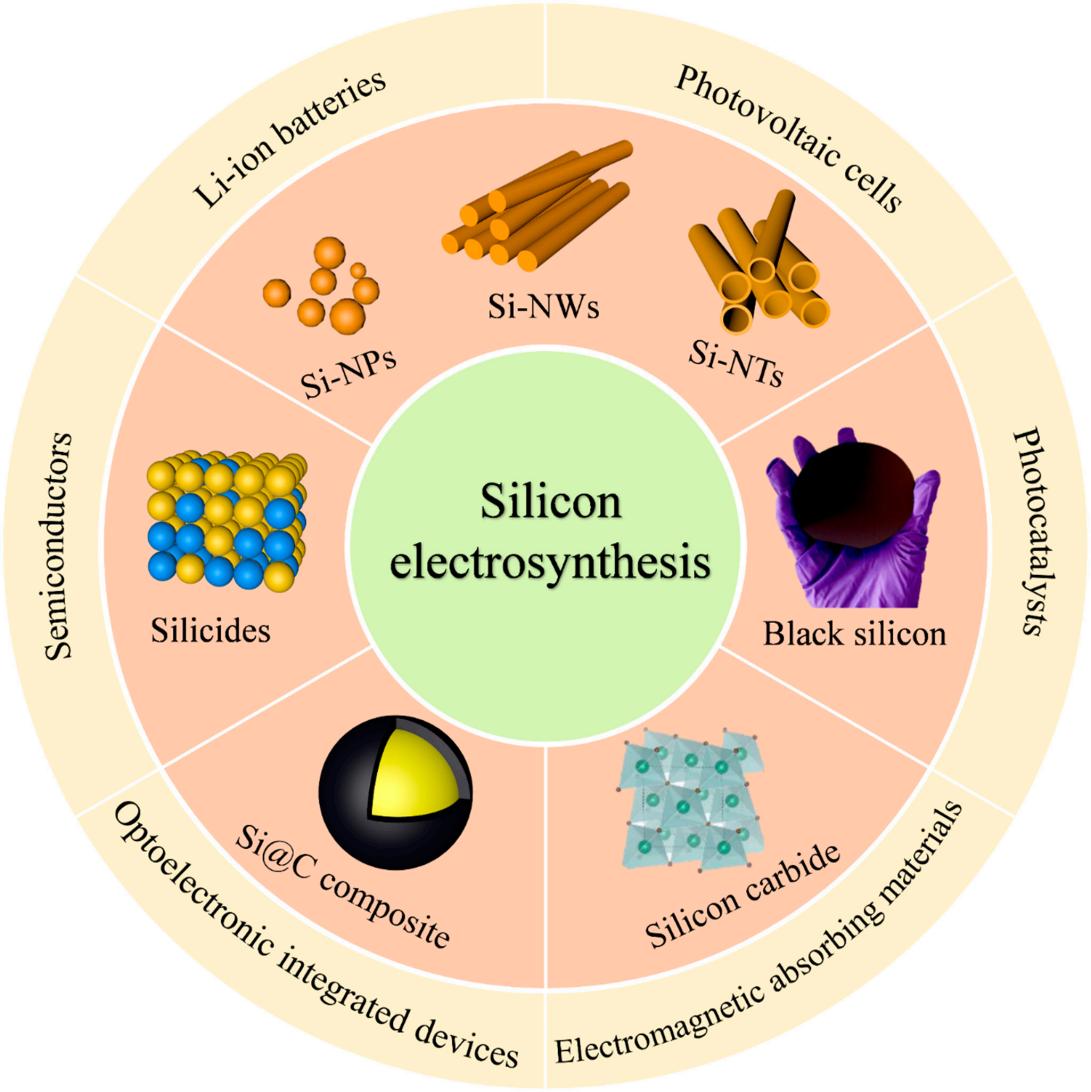
Schematic diagram of the fabrication of various silicon-based materials by molten salt electrolysis and their potential applications. Image of black silicon reproduced from [97] with the permission of the Royal Society of Chemistry.

The electrochemical strategy provides the feasibility of the design and synthesis of various silicon-based materials. Electro-deoxidation offers a simple and effective route for the synthesis of micro- and nanostructured silicon and other silicon compounds for many applications, including Li-ion batteries, PV cells, semiconductors, photocatalysts, integrated optoelectronic devices, and electromagnetic absorption materials (Fig. 17). Of particular concern is the application of silicon nanomaterials in Li-ion batteries, due to the fact that silicon has the highest specific capacity to store lithium ions. However, the severe structural degradation caused by the expansion–shrinkage of silicon during charging and discharging makes the lifetime of silicon-based cells difficult to meet. Thus, designing and creating multicomponent micro-nanostructures is an effective strategy to mitigate or suppress structural degradation of silicon anodes. The controlled fabrication of a broad range of nanostructures for silicon, such as Si-NP, Si-NW, and Si-NT, is now possible on a laboratory scale by electrochemical methods. Even more exciting is the fact that a pilot ton-scale production of silica-based anode materials for Li-ion batteries by electrolysis of silica in molten salt has been established in China [85]. Therefore, more emphasis should be placed on improving the electrochemical properties of silica-based anode materials based on this approach in the future. In addition, how the various electrolytic parameters for high-performance silica-based anodes achieved in laboratory scale can be transferred to industrial-scale production should be further explored. Moreover, future efforts should be aimed at expanding the electrochemical preparation of black silicon. This process is fairly simple and fast, avoiding the emission of toxic or corrosive chemicals or harmful substances. In addition, the direct extraction of SiC and Si/C composites by electrochemical methods from RH, an agricultural waste rich in silicon resources, should also attract more attention.

## References

[1] He T, Cao HJ, Chen P. Complex hydrides for energy storage, conversion, and utilization. Adv Mater. 2019;31(50): Article 1902757. 10.1002/adma.201902757 31682051

[2] Zhang SL, Liu Y, Fan QN, Zhang C, Zhou T, Kalantar-Zadeh K, Guo Z. Liquid metal batteries for future energy storage. Energy Environ Sci. 2021;14(8):4177–4202.

[3] Zu L, Zhang W, Qu L, Liu L, Li W, Yu A, Zhao D. Mesoporous materials for electrochemical energy storage and conversion. Adv Energy Mater. 2020;10(38): Article 2002152.

[4] Liu H, Zhang X, He SM, He D, Shang Y, Yu H. Molten salts for rechargeable batteries. Mater Today. 2022;60:128–157.

[5] Dai H, Wang L, Zhao Y, Xue J, Zhou R, Yu C, An J, Zhou J, Chen Q, Sun G, et al. Recent advances in molybdenum-based materials for lithium-sulfur batteries. Research. 2021;2021: Article 5130420. 33748762 10.34133/2021/5130420 PMC7949955

[6] Kyushin S, Kurosaki Y, Otsuka K, Imai H, Ishida S, Kyomen T, Hanaya M, Matsumoto H. Silicon-silicon π single bond. Nat Commun. 2020;11(1): Article 4009. 32782244 10.1038/s41467-020-17815-z PMC7419521

[7] Shirahata N, Nakamura J, Inoue J-i, Ghosh B, Nemoto K, Nemoto Y, Takeguchi M, Masuda Y, Tanaka M, Ozin GA. Emerging atomic energy levels in zero-dimensional silicon quantum dots. Nano Lett. 2020;20(3):1491–1498. 32046494 10.1021/acs.nanolett.9b03157

[8] Zhang MH, Yan TX, Wang W, Jia XX, Wang J, Klemes JJ. Energy-saving design and control strategy towards modern sustainable greenhouse: A review. Renew Sust Energ Rev. 2022;164: Article 112602.

[9] Andreani LC, Bozzola A, Kowalczewski P, Liscidini M, Redorici L. Silicon solar cells: Toward the efficiency limits. Adv Phys X. 2019;4(1): Article 1548305.

[10] Köhler M, Pomaska M, Procel P, Santbergen R, Zamchiy A, Macco B, Lambertz A, Duan W, Cao P, Klingebiel B, et al. A silicon carbide-based highly transparent passivating contact for crystalline silicon solar cells approaching efficiencies of 24%. Nat Energy. 2021;6(5):529–537.

[11] Xu LJ, Liu WZ, Liu HH, Ke C, Wang M, Zhang C, Aydin E, al-Aswad M, Kotsovos K, Gereige I, et al. Heat generation and mitigation in silicon solar cells and modules. Joule. 2021;5(3):631–645.

[12] Cui Y. Silicon anodes. Nat Energy. 2021;6(10):995–996.

[13] Eshetu GG, Zhang H, Judez X, Adenusi H, Armand M, Passerini S, Figgemeier E. Production of high-energy Li-ion batteries comprising silicon-containing anodes and insertion-type cathodes. Nat Commun. 2021;12(1):5459. 34526508 10.1038/s41467-021-25334-8 PMC8443554

[14] McBrayer JD, Rodrigues MTF, Schulze MC, Abraham DP, Apblett CA, Bloom I, Carroll GM, Colclasure AM, Fang C, Harrison KL, et al. Calendar aging of silicon-containing batteries. Nat Energy. 2021;6(9):866–872.

[15] Dong LW, Zhong SJ, Yuan BT, Ji Y, Liu J, Liu Y, Yang C, Han J, He W. Electrolyte engineering for high-voltage lithium metal batteries. Research. 2022;2022: Article 9837586. 36128181 10.34133/2022/9837586 PMC9470208

[16] Singh J, Agrahari A. The progression of silicon technology acting as substratum for the betterment of future photovoltaics. Int J Energy Res. 2019;43(9):3959–3980.

[17] Fu R, James TL, Woodhouse M. Economic measurements of polysilicon for the photovoltaic industry: Market competition and manufacturing competitiveness. IEEE J Photovolt. 2015;5(2):515–524.

[18] Louwen A, van Sark W, Schropp R, Faaij A. A cost roadmap for silicon heterojunction solar cells. Sol Energy Mater Sol Cells. 2016;147:295–314.

[19] Ye KK, Wang JQ, Xing PF, du X, Gao B, Kong J, Luo X. Study of the preparation of high purity silicon by a new electro-thermal metallurgy method. Silicon. 2019;11(3):1175–1184.

[20] Juzeliūnas E, Fray DJ. Silicon electrochemistry in molten salts. Chem Rev. 2020;120(3):1690–1709. 31886645 10.1021/acs.chemrev.9b00428

[21] Maldonado S. The importance of new “sand-to-silicon” processes for the rapid future increase of photovoltaics. ACS Energy Lett. 2020;5(11):3628–3632.

[22] Jiang TT, Xu XY, Chen GZ. Silicon prepared by electro-reduction in molten salts as new energy materials. J Energy Chem. 2020;47:46–61.

[23] Haraldsson J, Johansson MT. Review of measures for improved energy efficiency in production-related processes in the aluminium industry–from electrolysis to recycling. Renew Sust Energ Rev. 2018;93:525–548.

[24] Nohira T, Yasuda K, Ito Y. Pinpoint and bulk electrochemical reduction of insulating silicon dioxide to silicon. Nat Mater. 2003;2(6):397–401. 12754498 10.1038/nmat900

[25] Zein El Abedin S, Endres F. Ionic liquids: The link to high-temperature molten salts? Acc Chem Res. 2007;40(11):1106–1113. 17521159 10.1021/ar700049w

[26] Xu J, Haarberg GM. Electrodeposition of solar cell grade silicon in high temperature molten salts. High Temp Mater Process. 2013;32(2):97–105.

[27] Zhu GJ, Luo W, Wang LJ, Jiang W, Yang JP. Silicon: Toward eco-friendly reduction techniques for lithium-ion battery applications. J Mater Chem A. 2019;7(43):24715–24737.

[28] Dasog M, Kehrle J, Rieger B, Veinot JG. Silicon nanocrystals and silicon-polymer hybrids: Synthesis, surface engineering, and applications. Angew Chem Int Ed. 2016;55(7):2322–2339. 10.1002/anie.201506065 26607409

[29] Yasuda K, Nohira T. Electrochemical production of silicon. High Temp Mater Process. 2022;41(1):247–278.

[30] Elwell D, Feigelson RS. Electrodeposition of solar silicon. Sol Energy Mater Sol Cells. 1982;6(2):123–145.

[31] Elwell D, Rao GM. Electrolytic production of silicon. J Appl Electrochem. 1988;18(1):15–22.

[32] Monnier R, Giacometti JC. Recherches sur le raffinage électrolytique du silicium. Helv Chim Acta. 1964;47(2):345–353.

[33] Zhou QD, Kennedy BJ. High-temperature powder synchrotron diffraction studies of synthetic cryolite Na_3_AlF_6_. J Solid State Chem. 2004;177(3):654–659.

[34] Parker SF, Ramirez-Cuesta AJ, Daemen LL. The structure and vibrational spectroscopy of cryolite, Na_3_AlF_6_. RSC Adv. 2020;10(43):25856–25863. 35518579 10.1039/d0ra04804f PMC9055329

[35] Lu X, Zhang ZY, Hiraki T, Takeda O, Zhu H, Matsubae K, Nagasaka T. A solid-state electrolysis process for upcycling aluminium scrap. Nature. 2022;606:511–515. 35417651 10.1038/s41586-022-04748-4

[36] Monnier R. L’Obtention et le raffinage du silicium par voie electrochimique. Chimia. 1983;37(4): Article 109.

[37] Stubergh JR, Liu ZH. Preparation of pure silicon by electrowinning in a bytownite-cryolite melt. Metall Mater Trans B. 1996;27(6):895–900.

[38] Huggins RA, Elwell D. Morphological stability of a plane interface during electroncrystallization from molten salts. J Cryst Growth. 1977;37(2):159–162.

[39] De Mattei RC, Elwell D, Feigelson RS. Electrodeposition of silicon at temperatures above its melting point. J Electrochem Soc. 1981;128(8):1712–1714.

[40] Hossain MJ, Gregory G, Schneller EJ, Gabor AM, Blum AL, Yang Z, Sulas D, Johnston S, Davis KO. A comprehensive methodology to evaluate losses and process variations in silicon solar cell manufacturing. IEEE J Photovolt. 2019;9(5):1350–1359.

[41] Cohen U, Huggins RA. Silicon epitaxial growth by electrodeposition from molten fluorides. J Electrochem Soc. 1976;123(3):381–383.

[42] Rao GM, Elwell D, Feigelson RS. Electrowinning of silicon from K_2_SiF_6_-molten fluoride systems. J Electrochem Soc. 1980;127(9):1940–1944.

[43] Boen R, Bouteillon J. The electrodeposition of silicon in fluoride melts. J Appl Electrochem. 1983;13(3):277–288.

[44] De Lepinay J, Bouteillon J, Traore S, Renaud D, Barbier M. Electroplating silicon and titanium in molten fluoride media. J Appl Electrochem. 1987;17(2):294–302.

[45] Elwell D, Rao G. Mechanism of electrodeposition of silicon from K_2_SiF_6_-flinak. Electrochim Acta. 1982;27(6):673–676.

[46] Rao GM, Elwell D, Feigelson RS. Electrodeposition of silicon onto graphite. J Electrochem Soc. 1981;128(8):1708–1711.

[47] Bieber AL, Massot L, Gibilaro M, Cassayre L, Taxil P, Chamelot P. Silicon electrodeposition in molten fluorides. Electrochim Acta. 2012;62:282–289.

[48] Cai ZY, Li YG, Tian W. Electrochemical behavior of silicon compound in LiF–NaF–KF–Na_2_SiF_6_ molten salt. Ionics. 2011;17(9):821–826.

[49] Yasuda K, Maeda K, Nohira T, Hagiwara R, Homma T. Silicon electrodeposition in water-soluble KF-KCl molten salt: Optimization of electrolysis conditions at 923K. J Electrochem Soc. 2015;163(3):D95–D99.

[50] Maeda K, Yasuda K, Nohira T, Hagiwara R, Homma T. A new electrodeposition process of crystalline silicon utilizing water-soluble KF–KCl molten salt. ECS Trans. 2014;64(4):285.

[51] Yasuda K, Saeki K, Kato T, Hagiwara R, Nohira T. Silicon electrodeposition in a water-soluble KF-KCl molten salt: Effects of temperature and current density. J Electrochem Soc. 2018;165(16):D825–D831.

[52] Yasuda K, Kato T, Norikawa Y, Nohira T. Silicon electrodeposition in a water-soluble KF–KCl molten salt: Properties of Si films on graphite substrates. J Electrochem Soc. 2021;168(11): Article 112502.

[53] Peng JJ, Yin HY, Zhao J, Yang X, Bard AJ, Sadoway DR. Liquid-tin-assisted molten salt electrodeposition of photoresponsive n-type silicon films. Adv Funct Mater. 2018;28(1): Article 1703551.

[54] Laptev M, Isakov A, Grishenkova O, Vorob’ev AS, Khudorozhkova AO, Akashev LA, Zaikov YP. Electrodeposition of thin silicon films from the KF-KCl-KI-K_2_SiF_6_ melt. J Electrochem Soc. 2020;167(4): Article 042506.

[55] Laptev MV, Khudorozhkova AO, Isakov A, Grishenkova OV, Zhuk SI, Zaikov YP. Electrodeposition of aluminum-doped thin silicon films from a KF–KCl–KI–K_2_SiF_6_–AlF_3_ melt. J Serb Chem Soc. 2021;86(11):1075–1087.

[56] Krywko-Cendrowska A, Marot L, Strawski M, Steiner R, Meyer E. Electrodeposition and characterization of SiO*_x_* films photoactive in organic solution. J Electrochem Soc. 2015;163(3):D100–D106.

[57] Vichery C, Le Nader V, Frantz C, Zhang Y, Michler J, Philippe L. Stabilization mechanism of electrodeposited silicon thin films. Phys Chem Chem Phys. 2014;16(40):22222–22228. 25212513 10.1039/c4cp02797c

[58] Agrawal A, Austin A. Electrodeposition of silicon from solutions of silicon halides in aprotic solvents. J Electrochem Soc. 1981;128(11):2292–2296.

[59] Gobet J, Tannenberger H. Electrodeposition of silicon from a nonaqueous solvent. J Electrochem Soc. 1988;135(1):109–112.

[60] Nicholson J. Electrodeposition of silicon from nonaqueous solvents. J Electrochem Soc. 2005;152(12):C795–C802.

[61] Nishimura Y, Fukunaka Y. Electrochemical reduction of silicon chloride in a non-aqueous solvent. Electrochim Acta. 2007;53(1):111–116.

[62] Munisamy T, Bard AJ. Electrodeposition of Si from organic solvents and studies related to initial stages of Si growth. Electrochim Acta. 2010;55(11):3797–3803.

[63] Chen GZ, Fray DJ, Farthing TW. Direct electrochemical reduction of titanium dioxide to titanium in molten calcium chloride. Nature. 2000;407(6802):361–364. 11014188 10.1038/35030069

[64] Okabe TH, Park I, Jacob K, Waseda Y. Production of niobium powder by electronically mediated reaction (EMR) using calcium as a reductant. J Alloys Compd. 1999;288(1–2):200–210.

[65] Jin XB, Gao P, Wang DH, Hu XH, Chen GZ. Electrochemical preparation of silicon and its alloys from solid oxides in molten calcium chloride. Angew Chem Int Ed. 2004;43(6):733–736. 10.1002/anie.200352786 14755706

[66] Xiao W, Wang DH. The electrochemical reduction processes of solid compounds in high temperature molten salts. Chem Soc Rev. 2014;43(10):3215–3228. 24535552 10.1039/c3cs60327j

[67] Deng Y, Wang DH, Xiao W, Jin XB, Hu XH, Chen GZ. Electrochemistry at conductor/insulator/electrolyte three-phase interlines: A thin layer model. J Phys Chem B. 2005;109(29):14043–14051. 16852763 10.1021/jp044604r

[68] Xiao W, Jin XB, Deng Y, Wang DH, Hu XH, Chen GZ. Electrochemically driven three-phase interlines into insulator compounds: Electroreduction of solid SiO_2_ in molten CaCl_2_. ChemPhysChem. 2006;7(8):1750–1758. 16810659 10.1002/cphc.200600149

[69] Yasuda K, Nohira T, Amezawa K, Ogata YH, Ito Y. Mechanism of direct electrolytic reduction of solid SiO_2_ to Si in molten CaCl_2_. J Electrochem Soc. 2005;152(4):D69–D74.

[70] Xiao W, Jin XB, Deng Y, Wang DH, Chen GZ. Rationalisation and optimisation of solid state electro-reduction of SiO_2_ to Si in molten CaCl_2_ in accordance with dynamic three-phase interlines based voltammetry. J Electroanal Chem. 2010;639(1-2):130–140.

[71] Xiao W, Wang X, Yin HY, Zhu H, Mao XH, Wang DH. Verification and implications of the dissolution-electrodeposition process during the electro-reduction of solid silica in molten CaCl_2_. RSC Adv. 2012;2(19):7588–7593.

[72] Yang X, Ji L, Zou XL, Lim T, Zhao J, Yu ET, Bard AJ. Toward cost-effective manufacturing of silicon solar cells: Electrodeposition of high-quality Si films in a CaCl_2_-based molten salt. Angew Chem Int Ed. 2017;129(47):15274–15278. 10.1002/anie.201707635 28902971

[73] Zou XL, Ji L, Ge JB, Sadoway DR, Yu ET, Bard AJ. Electrodeposition of crystalline silicon films from silicon dioxide for low-cost photovoltaic applications. Nat Commun. 2019;10(1):5772. 31852891 10.1038/s41467-019-13065-w PMC6920409

[74] Kongstein OE, Wollan C, Sultana S, Haarberg GM. Electrorefining of silicon in molten calcium chloride. ECS Trans. 2007;3(35):357–361.

[75] Xu YZ, Hu XD, Kundu S, Nag A, Afsarimanesh N, Sapra S, Mukhopadhyay SC, Han T. Silicon-based sensors for biomedical applications: A review. Sensors. 2019;19(13): Article 2908. 31266148 10.3390/s19132908 PMC6651638

[76] Moss DJ, Morandotti R, Gaeta AL, Lipson M. New CMOS-compatible platforms based on silicon nitride and hydex for nonlinear optics. Nat Photonics. 2013;7(8):597–607.

[77] Atabaki AH, Moazeni S, Pavanello F, Gevorgyan H, Notaros J, Alloatti L, Wade MT, Sun C, Kruger SA, Meng H, et al. Integrating photonics with silicon nanoelectronics for the next generation of systems on a chip. Nature. 2018;556(7701):349–354. 29670262 10.1038/s41586-018-0028-z

[78] Sarai NS, Levin BJ, Roberts JM, Katsoulis DE, Arnold FH. Biocatalytic transformations of silicon—The other group 14 element. ACS Cent Sci. 2021;7(6):944–953. 34235255 10.1021/acscentsci.1c00182 PMC8227617

[79] Guo JP, Dong DQ, Wang J, Liu D, Yu X, Zheng Y, Wen Z, Lei W, Deng Y, Wang J, et al. Silicon-based lithium ion battery systems: State-of-the-art from half and full cell viewpoint. Adv Funct Mater. 2021;31(34): Article 2102546.

[80] Yang JY, Lu SG, Kan SR, Zhang XJ, Du J. Electrochemical preparation of silicon nanowires from nanometre silica in molten calcium chloride. Chem Commun. 2009;22:3273–3275. 10.1039/b902029b 19587937

[81] Xiao W, Jin XB, Chen GZ. Up-scalable and controllable electrolytic production of photo-responsive nanostructured silicon. J Mater Chem A. 2013;1(35):10243–10250.

[82] Zhao J, Li J, Ying PL, Zhang WH, Meng LJ, Li C. Facile synthesis of freestanding Si nanowire arrays by one-step template-free electro-deoxidation of SiO_2_ in a molten salt. Chem Commun. 2013;49(40):4477–4479. 10.1039/c3cc00101f 23571606

[83] Zhang J, Fang S, Qi XP, Yu Z, Wu Z, Yang J, Lu S. Preparation of high-purity straight silicon nanowires by molten salt electrolysis. J Energy Chem. 2020;40:171–179.

[84] Dong YF, Slade T, Stolt MJ, Li L, Girard SN, Mai L, Jin S. Low-temperature molten-salt production of silicon nanowires by the electrochemical reduction of CaSiO_3_. Angew Chem Int Ed. 2017;129(46):14645–14649. 10.1002/anie.201707064 28952181

[85] Yu ZL, Wang N, Fang S, Qi X, Gao Z, Yang J, Lu S. Pilot-plant production of high-performance silicon nanowires by molten salt electrolysis of silica. Ind Eng Chem Res. 2020;59(1):1–8.

[86] Weng W, Yang JR, Zhou J, Gu D, Xiao W. Template-free electrochemical formation of silicon nanotubes from silica. Adv Sci. 2020;7(17): Article 2001492. 10.1002/advs.202001492 PMC7507395 32995133

[87] Wang F, Liu W, Ma YS, Chen D, Li P, Yin H, Li W, Wang D. Fabricating silicon nanotubes by electrochemical exfoliation and reduction of layer-structured CaSiO_3_ in molten salt. ACS Appl Mater Interfaces. 2021;13(26):30668–30677. 34165965 10.1021/acsami.1c07031

[88] Wang F, Ma YS, Li P, Peng C, Yin H, Li W, Wang D. Electrochemical conversion of silica nanoparticles to silicon nanotubes in molten salts: Implications for high-performance lithium-ion battery anode. ACS Appl Nano Mater. 2021;4(7):7028–7036.

[89] Wang F, Li P, Li W, Wang DH. Electrochemical synthesis of multidimensional nanostructured silicon as a negative electrode material for lithium-ion battery. ACS Nano. 2022;16(5):7689–7700. 35445596 10.1021/acsnano.1c11393

[90] Jing SX, Xiao JX, Shen YJ, Hong B, Gu D, Xiao W. Silicate-mediated electrolytic silicon nanotube from silica in molten salts. Small. 2022;18(35): Article 2203251. 10.1002/smll.202203251 35934894

[91] Ivanova EP, Hasan J, Webb HK, Gervinskas G, Juodkazis S, Truong VK, Wu AHF, Lamb RN, Baulin VA, Watson GS, et al. Bactericidal activity of black silicon. Nat Commun. 2013;4:2838. 24281410 10.1038/ncomms3838 PMC3868328

[92] Savin H, Repo P, Von Gastrow G, Ortega P, Calle E, Garín M, Alcubilla R. Black silicon solar cells with interdigitated back-contacts achieve 22.1% efficiency. Nat Nanotechnol. 2015;10(7):624–628. 25984832 10.1038/nnano.2015.89

[93] Oh J, Yuan H-C, Branz HM. An 18.2%-efficient black-silicon solar cell achieved through control of carrier recombination in nanostructures. Nat Nanotechnol. 2012;7(11):743–748. 23023643 10.1038/nnano.2012.166

[94] Huo CL, Wang J, Fu HX, Li X, Yang Y, Wang H, Mateen A, Farid G, Peng KQ. Metal-assisted chemical etching of silicon in oxidizing HF solutions: Origin, mechanism, development, and black silicon solar cell application. Adv Funct Mater. 2020;30(52): Article 2005744.

[95] Juzeliunas E, Cox A, Fray DJ. Silicon surface texturing by electro-deoxidation of a thin silica layer in molten salt. Electrochem Commun. 2010;12(10):1270–1274.

[96] Juzeliunas E, Cox A, Fray DJ. Electro-deoxidation of thin silica layer in molten salt—Globular structures with effective light absorbance. Electrochim Acta. 2012;68:123–127.

[97] Coxon PR, Coto M, Juzeliunas E, Fray DJ. The use of electro-deoxidation in molten salts to reduce the energy consumption of solar grade silicon and increase the output of PV solar cells. Prog Nat Sci Mater Int. 2015;25(6):583–590.

[98] Cho SK, Fan F-RF, Bard AJ. Formation of a silicon layer by electroreduction of SiO_2_ nanoparticles in CaCl_2_ molten salt. Electrochim Acta. 2012;65:57–63.

[99] Cho SK, Fan FR, Bard AJ. Electrodeposition of crystalline and photoactive silicon directly from silicon dioxide nanoparticles in molten CaCl_2_. Angew Chem Int Ed. 2012;51(51):12740–12744. 10.1002/anie.201206789 23143938

[100] Zhao J, Yin HY, Lim T, Xie H, Hsu HY, Forouzan F, Bard AJ. Electrodeposition of photoactive silicon films for low-cost solar cells. J Electrochem Soc. 2016;163(9):D506–D514.

[101] Zou XL, Ji L, Yang X, Lim T, Yu ET, Bard AJ. Electrochemical formation of a *p–n* junction on thin film silicon deposited in molten salt. J Am Chem Soc. 2017;139(45):16060–16063. 29095608 10.1021/jacs.7b09090

[102] Chen X, Liang CH. Transition metal silicides: Fundamentals, preparation and catalytic applications. Catal Sci Technol. 2019;9(18):4785–4820.

[103] Hamaoui G, Horny N, Hua ZL, Zhu T, Robillard J-F, Fleming A, Ban H, Chirtoc M. Electronic contribution in heat transfer at metal-semiconductor and metal silicide-semiconductor interfaces. Sci Rep. 2018;8(1):11352. 30054516 10.1038/s41598-018-29505-4 PMC6063978

[104] Piva S, Pistorius PC. Ferrosilicon-based calcium treatment of aluminum-killed and silicomanganese-killed steels. Metall Mater Trans B. 2021;52(1):6–16.

[105] Imai M, Nishida K, Kimura T, Abe H. Superconductivity of Ca(Al_0.5_, Si_0.5_)_2_, a ternary silicide with the AlB_2_-type structure. Appl Phys Lett. 2002;80(6):1019–1021.

[106] Sakanaka Y, Goto T, Hachiya K. Electrochemical formation of ca-Si in molten CaCl_2_-KCl. J Electrochem Soc. 2015;162(4):D186–D191.

[107] Wang HL, Sun DK, Song QQ, Xie WQ, Jiang X, Zhang B. One-step electrolytic preparation of Si–Fe alloys as anodes for lithium ion batteries. Funct Mater Lett. 2016;9(03): Article 1650050.

[108] Gao Y, Peng MH, Sun Y, Yang Y, Wang XM. Preparation of MnSi alloy by direct electro-deoxidation from SiO_2_-MnO_2_ in molten salt for potential applications in lithium batteries. Optoelectron Adv Mater Rapid Commun. 2015;9(1–2):245–247.

[109] Zhou ZR, Zhang YJ, Hua YX, Dong P, Lin Y, Xu M, Wang D, Li X, Han L, Duan J. Molten salt electrolytic synthesis of silicon-copper composite nanowires with enhanced performances as lithium ion battery anode. J Alloys Compd. 2018;751:307–315.

[110] Zhou ZR, Dong P, Wang DY, Liu M, Duan J, Nayaka GP, Wang D, Xu C, Hua Y, Zhang Y. Silicon-titanium nanocomposite synthesized via the direct electrolysis of SiO_2_/TiO_2_ precursor in molten salt and their performance as the anode material for lithium ion batteries. J Alloys Compd. 2019;781:362–370.

[111] Cheng JX, Qiao JH, Yang ZY, Zhu B, Duan J, Wang D, Huang R, Zhang Y, Zhou Z, Dong P. Electrolytic preparation of porous TiSi_2_/Si nanocomposites and the electrochemical performances as lithium-ion battery anode. J Alloys Compd. 2022;890: Article 161732.

[112] Zou XL, Lu XG, Zhou ZF, Xiao W, Zhong Q, Li C, Ding W. Electrochemical extraction of Ti_5_Si_3_ silicide from multicomponent Ti/Si-containing metal oxide compounds in molten salt. J Mater Chem A. 2014;2(20):7421–7430.

[113] Xiao W, Zhou J, Yu L, Wang DH, Lou XW. Electrolytic formation of crystalline silicon/germanium alloy nanotubes and hollow particles with enhanced lithium-storage properties. Angew Chem Int Ed. 2016;128(26):7553–7557. 10.1002/anie.201602653 27159140

[114] Zou XL, Ji L, Pang ZY, Xu Q, Lu XG. Continuous electrodeposition of silicon and germanium micro/nanowires from their oxides precursors in molten salt. J Energy Chem. 2020;44:147–153.

[115] Tuci G, Liu Y, Rossin A, Guo X, Pham C, Giambastiani G, Pham-Huu C. Porous silicon carbide (SiC): A chance for improving catalysts or just another active-phase carrier? Chem Rev. 2021;121(17):10559–10665. 34255488 10.1021/acs.chemrev.1c00269

[116] Li F, Roccaforte F, Greco G, Fiorenza P, Via FL, Pérez-Tomas A, Evans JE, Fisher CA, Monaghan FA, Mawby PA, et al. Status and prospects of cubic silicon carbide power electronics device technology. Materials. 2021;14(19): Article 5831. 34640228 10.3390/ma14195831 PMC8510091

[117] Xu M, Girish YR, Rakesh KP, Wu P, Manukumar HM, Byrappa SM, Udayabhanu, Byrappa K. Recent advances and challenges in silicon carbide (SiC) ceramic nanoarchitectures and their applications. Mater Today Commun. 2021;28: Article 102533.

[118] Huang C, Zhang HC, Sun HD. Ultraviolet optoelectronic devices based on AlGaN-SiC platform: Towards monolithic photonics integration system. Nano Energy. 2020;77: Article 105149.

[119] Wang D, Huang L, Guo ZN, Han X, Liu C, Wang W, Yuan W. Enhanced photocatalytic hydrogen production over au/SiC for water reduction by localized surface plasmon resonance effect. Appl Surf Sci. 2018;456:871–875.

[120] Weng W, Zeng C, Xiao W. In situ pyrolysis concerted formation of Si/C hybrids during molten salt electrolysis of SiO_2_@polydopamine. ACS Appl Mater Interfaces. 2019;11(6):9156–9163. 30789694 10.1021/acsami.9b00265

[121] Cai MY, Zhou XB, Zhao ZQ, Ma Q, Xie H, Li X, Yin H. Engineering electrolytic silicon-carbon composites by tuning the in situ magnesium oxide space holder: Molten-salt electrolysis of carbon-encapsulated magnesium silicates for preparing lithium-ion battery anodes. ACS Sustain Chem Eng. 2020;8(26):9866–9874.

[122] Zhou XB, Xie HW, He X, Zhao Z, Ma Q, Cai M, Yin H. Annihilating the formation of silicon carbide: Molten salt electrolysis of carbon-silica composite to prepare the carbon-silicon hybrid for lithium-ion battery anode. Energy Environ Mater. 2020;3(2):166–176.

[123] Hossain SS, Mathur L, Roy P. Rice husk/rice husk ash as an alternative source of silica in ceramics: A review. J Asian Ceramic Soc. 2018;6(4):299–313.

[124] Wang ZF, Smith AT, Wang WX, Sun LY. Versatile nanostructures from rice husk biomass for energy applications. Angew Chem Int Ed. 2018;57(42):13722–13734. 10.1002/anie.201802050 29781126

[125] Kwofie E, Ngadi M. Sustainable energy supply for local rice parboiling in West Africa: The potential of rice husk. Renew Sust Energ Rev. 2016;56:1409–1418.

[126] Steven S, Restiawaty E, Bindar Y. Routes for energy and bio-silica production from rice husk: A comprehensive review and emerging prospect. Renew Sust Energ Rev. 2021;149: Article 111329.

[127] Akhter F, Soomro SA, Jamali AR, Chandio ZA, Siddique M, Ahmed M. Rice husk ash as green and sustainable biomass waste for construction and renewable energy applications: A review. Biomass Convers Biorefin. 2021;13:4639–4649.

[128] Zhao ZQ, Xie HW, Qu JK, Zhao H, Ma Q, Xing P, Song Q, Wang D, Yin H. A natural transporter of silicon and carbon: Conversion of rice husks to silicon carbide or carbon-silicon hybrid for lithium-ion battery anodes via a molten salt electrolysis approach. Batteries Supercaps. 2019;2(12):1007–1015.

[129] Pang D, Weng W, Zhou J, Gu D, Xiao W. Controllable conversion of rice husks to Si/C and SiC/C composites in molten salts. J Energy Chem. 2021;55:102–107.

[130] Weng W, Wang SB, Xiao W, Lou XW. Direct conversion of rice husks to nanostructured SiC/C for CO_2_ photoreduction. Adv Mater. 2020;32(29): Article 2001560. 10.1002/adma.202001560 32529684

[131] de Jesus SS, Maciel Filho R. Are ionic liquids eco-friendly? Renew Sust Energ Rev. 2022;157: Article 112039.

[132] Beil S, Markiewicz M, Pereira CS, Stepnowski P, Thöming J, Stolte S. Toward the proactive design of sustainable chemicals: Ionic liquids as a prime example. Chem Rev. 2021;121(21):13132–13173. 34523909 10.1021/acs.chemrev.0c01265

[133] Zhang T, Doert T, Wang H, Zhang SJ, Ruck M. Inorganic synthesis based on reactions of ionic liquids and deep eutectic solvents. Angew Chem Int Ed. 2021;60(41):22148–22165. 10.1002/anie.202104035 PMC8518931 34032351

[134] Katayama Y, Yokomizo M, Miura T, Kishi T. Preparation of a novel fluorosilicate salt for electrodeposition of silicon at low temperature. Electrochemistry. 2001;69(11):834–836.

[135] El Abedin SZ, Borissenko N, Endres F. Electrodeposition of nanoscale silicon in a room temperature ionic liquid. Electrochem Commun. 2004;6(5):510–514.

[136] Borisenko N, El Abedin SZ, Endres F. In situ STM investigation of gold reconstruction and of silicon electrodeposition on au(111) in the room temperature ionic liquid 1-butyl-1-methylpyrrolidinium bis(trifluoromethylsulfonyl)imide. J Phys Chem B. 2006;110(12):6250–6256. 16553441 10.1021/jp057337d

[137] Tsuyuki Y, Takai H, Fukunaka Y, Homma T. Formation of Si thin films by electrodeposition in ionic liquids for solar cell applications. Jpn J Appl Phys. 2018;57(8S3): Article 08RB11.

[138] Gu JS, Fahrenkrug E, Maldonado S. Direct electrodeposition of crystalline silicon at low temperatures. J Am Chem Soc. 2013;135(5):1684–1687. 23347180 10.1021/ja310897r

[139] Fahrenkrug E, Maldonado S. Electrochemical liquid-liquid-solid (ec-LLS) crystal growth: A low-temperature strategy for covalent semiconductor crystal growth. Acc Chem Res. 2015;48(7):1881–1890. 26132141 10.1021/acs.accounts.5b00158

[140] Zhang JL, Chen SM, Zhang HT, Zhang SJ, Yao X, Shi ZH. Electrodeposition of crystalline silicon directly from silicon tetrachloride in ionic liquid at low temperature. RSC Adv. 2016;6(15):12061–12067.

[141] Zhao ZX, Yang C, Wu L, Zhang CL, Wang RX, Ma E. Preparation and characterization of crystalline silicon by electrochemical liquid–liquid–solid crystal growth in ionic liquid. ACS Omega. 2021;6(18):11935–11942. 34056348 10.1021/acsomega.1c00304 PMC8154031

[142] Thomas S, Kowalski D, Molinari M, Mallet J. Role of electrochemical process parameters on the electrodeposition of silicon from 1-butyl-1-methylpyrrolidinium bis(trifluoromethanesulfonyl) imide ionic liquid. Electrochim Acta. 2018;265:166–174.

[143] Mallet J, Martineau F, Namur K, Molinari M. Electrodeposition of silicon nanotubes at room temperature using ionic liquid. Phys Chem Chem Phys. 2013;15(39):16446–16449. 23970072 10.1039/c3cp51522b

[144] Mallet J, Molinari M, Martineau F, Delavoie F, Fricoteaux P, Troyon M. Growth of silicon nanowires of controlled diameters by electrodeposition in ionic liquid at room temperature. Nano Lett. 2008;8(10):3468–3474. 18788792 10.1021/nl802352e

[145] Thomas S, Mallet J, Martineau F, Rinnert H, Molinari M. Strong room-temperature visible photoluminescence of amorphous Si nanowires prepared by electrodeposition in ionic liquids. ACS Photonics. 2018;5(7):2652–2660.

[146] Thomas S, Mallet J, Bahuleyan BK, Molinari M. Growth of homogeneous luminescent silicon-terbium nanowires by one-step electrodeposition in ionic liquids. Nanomaterials. 2020;10(12): Article 2390. 33265958 10.3390/nano10122390 PMC7760834

[147] Kowalski D, Mallet J, Thomas S, Nemaga AW, Michel J, Guery C, Molinari M, Morcrette M. Electrochemical synthesis of 1D core-shell Si/TiO_2_ nanotubes for lithium ion batteries. J Power Sources. 2017;361:243–248.

[148] Shen QY, Wang Q, Guo QJ, Guan SY, Li B. One-step electrodeposition of layer by layer architectural Si-graphene nanocomposite anode of lithium ion battery with enhanced cycle performance. J Electrochem Soc. 2018;165(3):D110–D115.

[149] Vanpariya A, Khanna S, Marathey P, Paneliya S, Mukhopadhyay I. Electrodeposition of silicon nanospheres on rGO coated copper substrate for lithium-ion batteries. Mater Today. 2021;47(Part 2):691–696.

[150] Li GM, Shen QX, Wang H, Guan SY, Li B. Alternative layered-structure SiCu composite anodes for high-capacity lithium-ion batteries. ACS Appl Energy Mater. 2022;5(1):740–749.

[151] Chen YF, Wang MY, Zhang JT, Tu JG, Ge JB, Jiao SQ. Green and sustainable molten salt electrochemistry for the conversion of secondary carbon pollutants to advanced carbon materials. J Mater Chem A. 2021;9(25):14119–14146.

[152] Yasuda K, Nohira T, Hagiwara R, Ogata YH. Direct electrolytic reduction of solid SiO_2_ in molten CaCl_2_ for the production of solar grade silicon. Electrochim Acta. 2007;53(1):106–110.

[153] Allanore A, Yin L, Sadoway DR. A new anode material for oxygen evolution in molten oxide electrolysis. Nature. 2013;497(7449):353–356. 23657254 10.1038/nature12134

[154] Du Y, Kou MY, Tu JG, Wang MY, Jiao SQ. An investigation into the anodic behavior of TiB_2_ in a CaCl_2_-based molten salt. Corros Sci. 2021;178: Article 109089.

[155] Jiao SQ, Fray DJ. Development of an inert anode for electrowinning in calcium chloride–calcium oxide melts. Metall Mater Trans B. 2010;41(1):74–79.

[156] Popescu A-M. Oxygen-evolving SnO_2_-based ceramic anodes in aluminium electrolysis. Chem Res Chin Univ. 2014;30(5):800–805.

[157] Mokkelbost T, Paulsen O, Xiao S, Haarberg GM, Ratvik AP. Fabrication and properties of SnO_2_-based inert gas anodes for electrowinning. ECS Trans. 2010;28(30):211–219.

[158] Jiao SQ, Zhang LL, Zhu HM, Fray DJ. Production of NiTi shape memory alloys via electro-deoxidation utilizing an inert anode. Electrochim Acta. 2010;55(23):7016–7020.

[159] Hu LW, Song Y, Ge JB, Jiao SQ, Cheng J. Electrochemical metallurgy in CaCl_2_-CaO melts on the basis of TiO_2_·RuO_2_ inert anode. J Electrochem Soc. 2015;163(3):E33–E38.

[160] Ge JB, Zou XL, Almassi S, Ji L, Chaplin BP, Bard AJ. Electrochemical production of Si without generation of CO_2_ based on the use of a dimensionally stable anode in molten CaCl_2_. Angew Chem Int Ed. 2019;58(45):16223–16228. 10.1002/anie.201905991 31483553

[161] Yasuda K, Maeda K, Hagiwara R, Homma T, Nohira T. Editors' Choice—Silicon electrodeposition in a water-soluble KF–KCl molten salt: Utilization of SiCl_4_ as Si source. J Electrochem Soc. 2017;164(2):D67–D71.

[162] Yasuda K, Shimao T, Hagiwara R, Homma T, Nohira T. Electrolytic production of silicon using liquid zinc alloy in molten CaCl_2_. J Electrochem Soc. 2017;164(8):H5049–H5056.

[163] Sofia SE, Mailoa JP, Weiss DN, Stanbery BJ, Buonassisi T, Peters IM. Economic viability of thin-film tandem solar modules in the United States. Nat Energy. 2018;3(5):387–394.

[164] Yamaguchi M, Masuda T, Araki K, Sato D, Lee KH, Kojima N, Takamoto T, Okumura K, Satou A, Yamada K, et al. Development of high-efficiency and low-cost solar cells for PV-powered vehicles application. Prog Photovolt. 2021;29(7):684–693.

[165] Smith BL, Woodhouse M, Horowitz KA, Silverman TJ, Zuboy J, Margolis RM. Photovoltaic (PV) module technologies: 2020 benchmark costs and technology evolution framework results (No. NREL/TP-7A40-78173, National Renewable Energy Laboratory, 2021).

